# Identifying and ranking causal biochemical biomarkers for breast cancer: a Mendelian randomisation study

**DOI:** 10.1186/s12916-022-02660-2

**Published:** 2022-11-23

**Authors:** Sonja N. Tang, Verena Zuber, Konstantinos K. Tsilidis

**Affiliations:** 1 Department of Epidemiology and Biostatistics, School of Public Health, Imperial College London, London, UK; 2 MRC Centre for Environment and Health, School of Public Health, Imperial College London, London, UK; 3 UK Dementia Research Institute at Imperial College, Imperial College London, London, UK; 4 Department of Hygiene and Epidemiology, University of Ioannina School of Medicine, Ioannina, Greece

**Keywords:** Mendelian randomisation, Breast cancer, Biomarkers, Instrumental variables, Causal inference, Epidemiology

## Abstract

**Background:**

Only a few of the 34 biochemical biomarkers measured in the UK Biobank (UKB) have been associated with breast cancer, with many associations suffering from possible confounding and reverse causation. This study aimed to screen and rank all UKB biochemical biomarkers for possible causal relationships with breast cancer.

**Methods:**

We conducted two-sample Mendelian randomisation (MR) analyses on ~420,000 women by leveraging summary-level genetic exposure associations from the UKB study (*n* = 194,174) and summary-level genetic outcome associations from the Breast Cancer Association Consortium (*n* = 228,951). Our exposures included all 34 biochemical biomarkers in the UKB, and our outcomes were overall, oestrogen-positive, and oestrogen-negative breast cancer. We performed inverse-variance weighted MR, weighted median MR, MR-Egger, and MR-PRESSO for 30 biomarkers for which we found multiple instrumental variables. We additionally performed multivariable MR to adjust for known risk factors, bidirectional MR to investigate reverse causation, and MR Bayesian model averaging to rank the significant biomarkers by their genetic evidence.

**Results:**

Increased genetic liability to overall breast cancer was robustly associated with the following biomarkers by decreasing importance: testosterone (odds ratio (OR): 1.12, 95% confidence interval (CI): 1.04–1.21), high-density lipoprotein (HDL) cholesterol (OR: 1.08, 95% CI: 1.04–1.13), insulin-like growth factor 1 (OR: 1.08, 95% CI: 1.02–1.13), and alkaline phosphatase (ALP) (OR: 0.93, 95% CI: 0.89–0.98).

**Conclusions:**

Our findings support a likely causal role of genetically predicted levels of testosterone, HDL cholesterol, and IGF-1, as well as a novel potential role of ALP in breast cancer aetiology. Further studies are needed to understand full disease pathways that may inform breast cancer prevention.

**Supplementary Information:**

The online version contains supplementary material available at 10.1186/s12916-022-02660-2.

## Background

Breast cancer is the most common cancer in women, with the lifetime risk of breast cancer for women in highly economically developed countries being 1 in 9. While breast cancer is a leading cause of death in women [1], the exact mechanisms of breast cancer initiation and progression are not known [2], necessitating a better understanding of disease aetiology.

The UK Biobank (UKB) study is a prospective cohort study that measured the genotypes and levels of 34 biochemical biomarkers of around 500,000 participants aged between 40 and 69 years, of which we sampled 194,174 women of white-British ancestry [3]. The biomarkers are grouped into six categories, namely bone and joint, cancer, cardiovascular, diabetes, liver, and renal biomarkers, which were measured due to their established relevance in a range of diseases and their diagnostic value and because they characterise phenotypes that are otherwise difficult to assess.

A few observational studies have been performed to study the associations between some of the UKB biochemical biomarkers and overall breast cancer, and significant associations have been found for several biomarkers. However, observational studies are prone to residual confounding and reverse causation. Mendelian randomisation (MR) complements observational studies by using genetic variants as instrumental variables (IVs) to establish likely causal associations between exposures and outcomes. To our knowledge, fewer than half of the biochemical biomarkers in the UKB have been investigated for likely causal associations with overall breast cancer using MR, and even fewer studies have stratified breast cancer by oestrogen receptor (ER) presence, which influences the disease prognosis and type of therapy that will be most effective [2]. See Additional file 1: Table S1 for a summary of the most recent observational and MR findings associating the UKB biochemical biomarkers with breast cancer in the literature.

This study aimed to use an MR framework to (1) explore univariable associations between genetically predicted levels of UKB biochemical biomarkers and genetic liability to overall, ER-positive, and ER-negative breast cancer; (2) investigate significant associations in detail through multivariable and bidirectional approaches; and (3) to rank the associated biomarkers by genetic evidence using a multivariable Bayesian MR approach. We achieved our aims by replicating and extending previous analyses to a bigger sample containing ~420,000 women and providing novel evidence for biomarkers not previously studied using MR.

## Methods

### Analysis plan

Our prospective plan was to carry out a variety of two-sample univariable MR (UVMR) analyses to examine the associations of each of the UKB biochemical biomarkers with overall, ER-positive, and ER-negative breast cancer liability. After our UVMR analyses showed significant associations, we performed further multivariable MR (MVMR) analyses to adjust for known risk factors and bidirectional analyses. We finally ranked our nominally significant biomarkers by importance using a multivariable Bayesian approach [4]. Our analysis follows the guidelines for performing MR investigations [5] and our reporting follows the guidelines for strengthening the reporting of Mendelian randomization studies (STROBE-MR) (Additional file 2: Checklist S1) [6]. We did not pre-register the study protocol.

### Study populations

Our study used summary-level exposure data from the UKB study [7] and summary-level outcome data from the Breast Cancer Association Consortium (BCAC) [8]. The BCAC includes ~6000 samples from the UK [8], which amounts to, at most, a ~1.4% sample overlap between the exposure and outcome samples. Our data only includes women of European descent to reduce bias from population stratification.

#### Exposure data

We obtained publicly available summary-level genome-wide association study (GWAS) statistics on 34 serum, urine, and red blood cell biomarker levels; body mass index (BMI); and alcohol intake frequency from unrelated female participants of white-British ancestry (*n* = 194,174) in the UKB cohort study from Neale et al. [9]. The genotypes and 34 biomarker levels were collected by the UKB study at baseline between 2006 and 2010 using various laboratory techniques and instruments by different suppliers [7, 10]. The GWASes were performed using age, age^2, and the first 20 principal components (PCs) as covariates [11]. Inverse-rank normalised GWAS data was used because many of the quantitative biomarker traits were non-normally distributed. Most women (at least 59%) in the UKB cohort were post-menopausal [12]. More information about the panel of UKB biomarkers and the original UKB study can be found elsewhere [3, 7].

#### Outcome data

Publicly available GWAS summary statistics on overall breast cancer cases (*n* = 122,977) and controls (*n* = 105,974) of European ancestry were obtained from the BCAC [13]. Of the breast cancer cases, 69,501 were ER-positive, 21,468 were ER-negative, and the majority developed post-menopause. More details about the original studies are described elsewhere [8, 14, 15].

### Statistical analysis

#### Selection of instrumental variables

For each exposure, we selected associated single-nucleotide polymorphisms (SNPs) at genome-wide significance (*P* < 5 × 10^−8^) and ensured their independence by removing those in linkage disequilibrium using the PLINK method (*r* ^2^ < 0.001, clumping distance = 10,000kb). We then harmonised the directions of the effect alleles between exposures and outcomes.

In all our MR analyses, SNPs must satisfy three assumptions to be considered valid IVs. Genetic variants must (1) strongly associate with the exposure (the relevance assumption), (2) be independent of confounders (the independence assumption), and (3) affect the outcome only through their effect on the exposure (the exclusion restriction assumption).

#### Univariable analyses

The main univariable analysis consisted of inverse-variance weighted (IVW) MR between each exposure and each outcome. The IVW method first estimates the Wald ratio for each SNP by dividing the SNP-outcome association by the SNP-exposure association and then combines these ratios in a fixed effect meta-analysis where each ratio is weighted by the inverse of the variance of the SNP-outcome association [16]. We used *P* < 0.05 as the nominal significance threshold. We also derived false discovery rate (FDR)-corrected *P*-values with the Benjamini-Hochberg (BH) method and used *P* < 0.05 as the FDR-corrected significance threshold. For exposures for which only 1 IV could be identified, we estimated the Wald ratio [17]. Our results are reported as odds ratios (OR) per standard deviation (SD) change in the genetically predicted biomarker concentration.

A common violation of the exclusion restriction IV condition is caused by horizontal pleiotropy, where a genetic variant has an effect on the outcome that does not occur through the exposure [18]. Therefore, we employed several additional univariable approaches with different underlying assumptions about the structure of the pleiotropy for all exposures, including the MR-Egger [19], weighted median [20], and MR Pleiotropy RESidual Sum and Outlier (MR-PRESSO) [21]. The MR-Egger allows for some directional pleiotropy in its estimate of the causal effect by making the additional Instrument Strength Independent of Direct Effect (InSIDE) assumption, which states that across all instruments, the magnitude of the pleiotropic effect is independent of the strength of the genetic variant-exposure association [19]. The weighted median allows for sparse or balanced pleiotropy by down-weighting outliers [20]. The MR-PRESSO method allows for some directional pleiotropy by identifying and adjusting for outliers [21].

#### Sensitivity analyses

We tested the robustness of our univariable findings by performing MVMR [22, 23] and bidirectional MR. MVMR was used to adjust for previously reported risk factors, while bidirectional MR was employed to rule out potential reverse causation.

We performed two-sample MVMR analyses for all seven biomarkers that were nominally significantly associated with overall breast cancer in IVW MR. We searched for associations at *P* < 10^−8^ of all variants used as IVs in Phenoscanner [24, 25] (Additional file 3: T1-T7), a database providing summarised GWASes, and adjusted for traits that could be considered reasons for horizontal pleiotropy. MVMR assumes that pleiotropic pathways operate through the risk factors included in the model [18]. For all MVMR analyses, we included SNPs that were genome-wide significantly associated (*P* < 5 × 10^−8^) with any exposure or risk factor that was taken into consideration in an MVMR model and not in linkage disequilibrium (*r* ^2^ < 0.001, clumping distance = 10,000kb).

As lipids are correlated [26], we included HDL cholesterol, low-density lipoprotein (LDL) cholesterol, triglycerides, and lipoprotein A in MVMR models to observe the direct associations of each lipid with each outcome.

As BMI [27] and alcohol intake [28] are associated with breast cancer risk, we included BMI and alcohol intake frequency in MVMR models for each of the seven biomarkers that we found to be nominally significantly associated with overall breast cancer in IVW MR.

As oestrogen decreases alkaline phosphatase (ALP) expression and activity in breast cancer cells [29] and we could not obtain enough genetic variants for oestradiol, we adjusted for testosterone and SHBG in an MVMR model with ALP.

After adjusting for BMI in MVMR, significant associations between SHBG and breast cancer risk have been found [28], so [30] we included BMI and SHBG in MVMR models.

Due to the low prior probability of association between ALP and breast cancer, we performed a bidirectional univariable MR analysis of genetically predicted overall, ER-positive, and ER-negative breast cancer liability and ALP levels.

#### Exposure rankings

We used MR Bayesian model averaging (MR-BMA) to agnostically rank the causal importance of the seven biomarkers found to be nominally significantly associated with overall breast cancer in IVW MR while accounting for potential pleiotropy [4]. Empirical *P*-values were calculated using a permutation approach [31] and adjusted for multiple testing using the BH method with *P* < 0.05 as the significance threshold. All independent (*r* ^2^ < 0.001) genetic variants associated with any of the biomarkers at genome-wide significance were included in the analysis (*n* = 460).

We used MR-BMA to consider each combination of biomarkers (all single biomarkers, all pairs of biomarkers, all triplets, and so on) as a candidate model in an MVMR analysis using weighted regression. Each candidate model was assigned a posterior probability (PP) that expresses the likelihood that the candidate model contains the true set of causal biomarkers using the regression’s goodness-of-fit measure.

Then, we used MR-BMA to perform model-averaging to assign each biomarker a marginal inclusion probability (MIP) and report each biomarker’s model-averaged causal effect (MACE) on overall breast cancer. The MIP represents the probability that the biomarker is a causal determinant of breast cancer risk, and the MACE represents the biomarker’s weighted average direct causal effect on risk across all candidate models. The MIP was calculated by summing up the posterior probabilities of all candidate models where the biomarker is present. The MACE underestimates the true causal effect of a biomarker on overall breast cancer and should not be interpreted in absolute terms, but as an indication of the effect direction and to compare the relative causal effects among biomarkers.

We used 0.5 as the prior probability for inclusion in the main analysis, which reflected an a priori belief that half of the candidate models or that half of the nominally significant biomarkers are causal, and priors of 0.25 and 0.75 as sensitivity analyses.

### Software

We employed the TwoSampleMR [31], MendelianRandomization [32], MRPRESSO [33], and ieugwasr [34] R packages, as well as the GitHub repository https://github.com/verena-zuber/ for MR-BMA for our analyses using R (version 4.0.5). We searched for secondary trait associations using Phenoscanner [24, 25].

## Results

### Overall breast cancer results

#### Univariable analyses

**Fig. 1:**
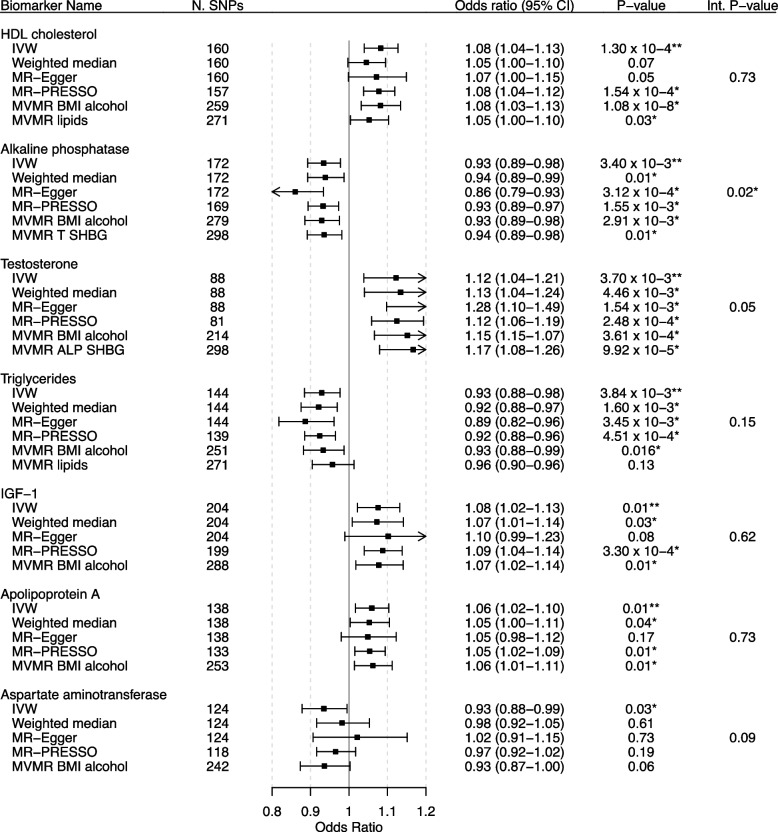
MR forest plot of significant biomarkers on overall breast cancer liability. Biomarkers of nominal significance in IVW MR analyses are shown in descending order of significance. The forest plot in the centre displays the odds ratio of the effect of an SD increase in genetically predicted concentration on overall breast cancer liability as a square, with error bars representing the 95% CI. In addition to the main analysis based on IVW MR, we include sensitivity analyses based on the weighted median, MR-Egger, MR-PRESSO, and MVMR accounting for known pleiotropic pathways. N. SNPs, number of SNPs; CI, confidence interval; Int. P-value, intercept *P*-value of MR-Egger; T, testosterone; BMI, body mass index. An asterisk (*) indicates nominal significance. Two asterisks (**) indicate FDR-corrected significance

We screened all UKB biochemical biomarkers for likely causal associations with overall breast cancer using various univariable MR methods. Using IVW MR, we found multiple testing adjusted significant associations of genetically predicted levels of six biomarkers and nominally significant associations of seven biomarkers and overall breast cancer liability. The IVW MR ORs and confidence intervals (CI) for a SD increase in the genetically predicted biomarker levels were the following: HDL cholesterol (OR: 1.08, 95% CI: 1.04–1.13), ALP (OR: 0.93, 95% CI: 0.89–0.98), testosterone (OR: 1.12, 95% CI: 1.04–1.21), triglycerides (OR: 0.93, 95% CI: 0.88–0.98), IGF-1 (OR: 1.08, 95% CI: 1.02–1.13), and apolipoprotein A (ApoA) (OR: 1.06, 95% CI: 1.02–1.10). Genetically predicted aspartate aminotransferase was nominally significantly associated with overall breast cancer liability in IVW MR (OR: 0.93, 95% CI: 0.88–0.99). We used an average of 147 SNPs with *F*-statistics ranging from 29 to 2360 as IVs for IVW analyses for these biomarkers (Additional file 3: T8-T14). *F* > 10 is considered the threshold for a strong instrument [18]. For these biomarkers, the weighted median, MR-Egger, and MR-PRESSO effect directions and sizes were largely consistent with our IVW MR findings, apart from the MR-Egger result for aspartate aminotransferase (Fig. 1).

While genetically predicted C-reactive protein levels were not significantly associated with overall breast cancer liability in the IVW MR, they were nominally significantly positively associated in the MR-Egger (OR: 1.12, 95% CI: 1.01–1.23) and MR-PRESSO (OR: 1.07, 95% CI: 1.02–1.11). We found no evidence of associations between genetically predicted levels of any of the remaining 27 UKB biomarkers and overall breast cancer liability (Additional file 4: Figs. S1-S6).

A summary of the findings for all 34 biomarkers in the context of the existing literature can be found in Additional file 5: Table S1.

#### Sensitivity analyses

After adjusting for LDL cholesterol, triglycerides, and lipoprotein A in MVMR, genetically predicted HDL cholesterol remained nominally significantly associated with overall breast cancer liability (OR: 1.05, 95% CI: 1.00–1.10) (Additional file 4: Fig. S7).

After adjusting for BMI and alcohol in MVMR, genetically predicted HDL cholesterol, ALP, testosterone, triglycerides, IGF-1, and apoA had significant direct effects on overall breast cancer liability, while aspartate aminotransferase did not (Additional file 4: Fig. S8).

Genetically predicted alkaline phosphatase remained nominally significantly associated with overall breast cancer liability after adjusting for testosterone and SHBG in MVMR (OR: 0.94, 95% CI: 0.89–0.98) (Additional file 4: Fig. S9).

We found no evidence of an association between genetically predicted SHBG and overall breast cancer liability after adjusting for BMI in MVMR (Additional file 4: Fig. S10).

We found no evidence of an association in bidirectional MR between genetically predicted overall breast cancer liability and genetically predicted ALP concentrations (Additional file 1: Table S2).

#### Exposure rankings

We used MR-BMA to rank the seven genetically predicted biomarkers that were nominally significantly associated with overall breast cancer liability in the IVW MR according to their MIP and with a prior probability of inclusion of 0.5. The biomarkers in the ranking showing the strongest evidence of causality with FDR-corrected significant *P*-values were testosterone (MIP = 0.979), HDL cholesterol (MIP = 0.704), IGF-1 (MIP = 0.639), and ALP (MIP = 0.583) (Table 1). The MACE directions of these biomarkers also exhibited consistency with our IVW MR results. Sensitivity analyses with priors of 0.25 and 0.75 did not impact the overall rankings (Additional file 1: Tables S3-S4).

**Table 1:** An MR-BMA ranking of individual biomarkers according to their marginal inclusion probability with a prior probability of inclusion of 0.5 for overall breast cancer

	Exposure	Marginal inclusion probability	Model-averaged causal effect	***P***-value	FDR
1	Testosterone	0.979	0.079	0.001*	0.006*
2	HDL cholesterol	0.704	0.035	0.002*	0.006*
3	IGF-1	0.639	0.025	0.017*	0.032*
4	Alkaline phosphatase	0.583	−0.019	0.018*	0.032*
5	Apolipoprotein A	0.224	−0.010	0.125	0.175
6	Triglycerides	0.064	−0.001	0.868	0.987
7	Aspartate aminotransferase	0.032	0	0.987	0.987

A ranking of the seven biomarkers nominally significantly associated with overall breast cancer in IVW MR. FDR; false discovery rate. An asterisk (*) indicates nominal significance

We also ranked our candidate models according to their PP with a prior of 0.5 (Additional file 1: Table S5), and we observed a high probability of testosterone, HDL cholesterol, IGF-1, and ALP being included in all candidate models. Sensitivity analyses with priors of 0.25 and 0.75 indicated consistent results (Additional file 1: Tables S6-S7).

### Results stratified by oestrogen receptor (ER) status

#### Univariable analyses

**Fig. 2:**
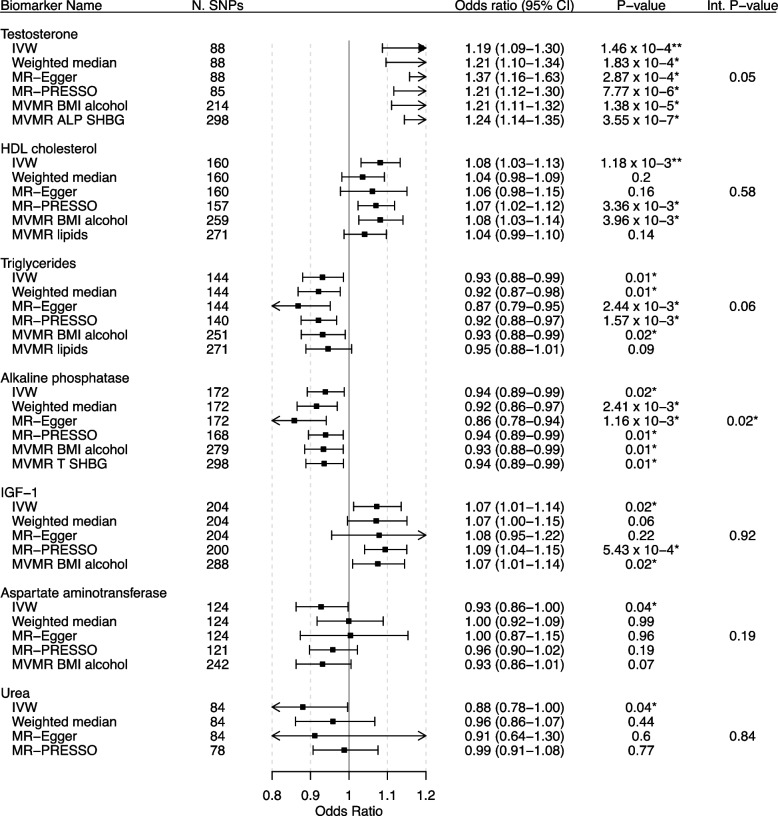
MR forest plot of significant biomarkers on ER-positive breast cancer liability. Biomarkers of nominal significance in IVW MR analyses are shown in descending order of significance. The forest plot in the centre displays the odds ratio of the effect of a SD increase in genetically predicted biomarker concentration on overall breast cancer liability as a square, with error bars representing the 95% CI. In addition to the main analysis based on IVW MR, we include sensitivity analyses based on the weighted median, MR-Egger, MR-PRESSO, and MVMR accounting for known pleiotropic pathways. N. SNPs, number of SNPs; CI, confidence interval; Int. P-value, intercept *P*-value of MR-Egger; T, testosterone; BMI, body mass index. An asterisk (*) indicates nominal significance. Two asterisks (**) indicate FDR-corrected significance

We stratified the outcome by ER status and screened all UKB biochemical biomarkers for likely causal associations using various univariable MR methods and found multiple testing adjusted significant associations between genetically predicted levels of testosterone (OR: 1.19, 95% CI: 1.09–1.30) and HDL cholesterol (OR: 1.08, 95% CI: 1.03–1.13), as well as nominally significant associations of triglycerides (OR: 0.93, 95% CI: 0.88–0.99), ALP (0.94, 0.89–9.99), IGF-1 (OR: 1.07, 95% CI: 1.01–1.14), aspartate aminotransferase (OR: 0.93, 95% CI: 0.86–1.00), and urea (OR: 0.88, 95% CI: 0.78–1.00) with ER-positive breast cancer liability in IVW MR (Fig. 2). We found consistent effect directions across the weighted median, MR-Egger, and MR-PRESSO analyses for these biomarkers apart from genetically predicted aspartate aminotransferase and urea. Weighted median, MR-Egger, and MR-PRESSO analyses showed nominally significant positive associations between genetically predicted C-reactive protein (CRP) and ER-positive breast cancer liability (Additional file 4: Fig. S11). We found no evidence of associations between any of the 27 remaining UKB biomarkers and ER-positive breast cancer liability (Additional file 4: Figs. S11-16).

**Fig. 3:**
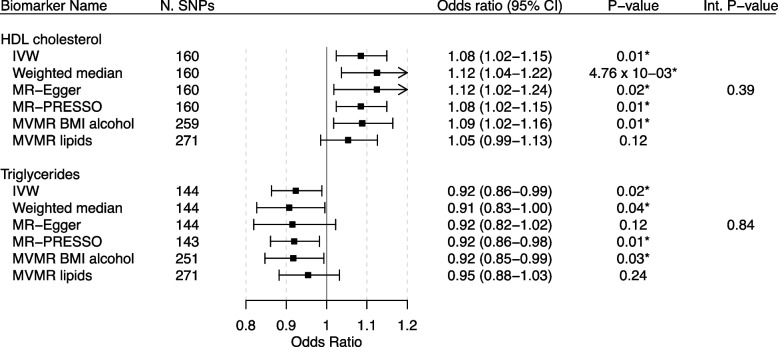
MR forest plot of significant biomarkers on ER-negative breast cancer liability. Biomarkers of nominal significance in IVW MR analyses are shown in descending order of significance. The forest plot in the centre displays the odds ratio of the effect of a SD increase in genetically predicted biomarker concentration on overall breast cancer liability as a square, with error bars representing the 95% CI. In addition to the main analysis based on IVW MR, we include sensitivity analyses based on the weighted median, MR-Egger, MR-PRESSO, and MVMR accounting for known pleiotropic pathways. N. SNPs, number of SNPs; CI, confidence interval; Int. P-value, intercept *P*-value of MR-Egger; BMI, body mass index. An asterisk (*) indicates nominal significance. Two asterisks (**) indicate FDR-corrected significance

We found nominally significant associations of genetically predicted levels of HDL cholesterol (OR: 1.08, 95% CI: 1.02–1.15) and triglycerides (OR: 0.92, 95% CI: 0.86–0.99) with ER-negative breast cancer liability in IVW MR (Fig. 3). We found no evidence of associations between any of the 32 remaining UKB biomarkers and ER-negative breast cancer liability (Additional file 4: Figs. S17-22).

#### Sensitivity analyses

We found no evidence of associations of lipids with ER-positive or ER-negative breast cancer liability in MVMR models (Additional file 4: Figs. S23 and S24).

We included each of the seven biomarkers that were nominally significantly associated with overall breast cancer in IVW MR in MVMR models with BMI and alcohol. Genetically predicted ALP, HDL cholesterol, IGF-1, testosterone, and triglycerides nominally significant direct associations with ER-positive breast cancer liability, while apoA, HDL cholesterol, and triglycerides had nominally significant direct associations with ER-negative breast cancer liability (Additional file 4: S25 and S26).

After adjusting for testosterone and SHBG in MVMR, genetically predicted ALP remained significantly associated with ER-positive breast cancer liability and we continued seeing no evidence of association with ER-negative breast cancer liability (Additional file 4: Figs. S27 and S28).

After adjusting for BMI in MVMR, genetically predicted SHBG was significantly associated with ER-positive, but not with ER-negative breast cancer liability (Additional file 4: Figs. S29 and S30).

We found no evidence of association in the bidirectional MR between genetically predicted ER-positive or ER-negative breast cancer liability and ALP levels (Additional file 1: Tables S8-S9 Tables).

## Discussion

In this study, we used a hypothesis-generating two-sample summary-level MR approach to screen the UKB for biochemical breast cancer biomarkers. We found that increases of 1 standard deviation in the genetically predicted levels of testosterone, HDL cholesterol, IGF-1, and ALP were robustly and consistently associated with overall breast cancer liability in a variety of univariable, multivariable, bidirectional, and ranking methods based on MR. These associations remained for ER-positive breast cancer, but only HDL cholesterol remained associated with ER-negative breast cancer. To our knowledge, ALP has not been associated with breast cancer before. The summary of our findings and how these compared with the literature to the best of our knowledge can be found in Additional file 5: Table S1.

For bone and joint biomarkers, we observed a novel inverse association between genetically predicted levels of ALP and overall and ER-positive breast cancer liability that was robust in all MR analyses. One possible explanation for this finding is that ALP-prioritised genes are enriched in primary and secondary sexual organs, and crucially, gene sets enriched among ALP-associated variants included oestradiol 17-beta-dehydrogenase activity, which catalyses oestradiol to the less potent estrone, thus reducing the risk of breast cancer [35]. We were unable to adjust our findings for oestradiol concentrations, as there are no large, high-quality GWASes for oestradiol. We instead adjusted for testosterone and SHBG in MVMR and did not observe an attenuation of the effect. Future research is required to clarify whether the ALP and breast cancer liability association is independent of oestrogens. A nominally significant negative association between serum levels of calcium and overall breast cancer risk was found in cohort studies [36], but not in an MR study [37], with which our study concurs. While vitamin D is negatively associated in observational analyses, no evidence of association could be found in MR [38], in agreement with the current study. We found no evidence of an association between genetically predicted rheumatoid factor and breast cancer liability.

For cancer biomarkers, a previous observational and MR study found positive associations between levels of IGF-1 and overall breast cancer risk in women in the UKB [39], which agrees with our findings. IGF-1 has long been implicated in breast cancer due to the role of IGF-1 receptors in activating the AKT and mitogen-activated protein kinase signalling networks in tumour growth [40]. A meta-analysis of observational studies found positive associations between oestradiol and overall breast cancer risk in post-menopausal women [41], which we could not confirm or dispute due to a lack of valid IVs for our MR analyses. This was likely due to imprecise measurements of oestradiol levels in the UKB [42], which was also a problem in a different study that led to oestradiol being excluded [43]. Positive associations between testosterone and breast cancer were found in a meta-analysis of prospective studies [41] and an MR study [42], which agree with our study. One hypothesis for our observed positive association of genetically predicted testosterone with overall, and ER-positive, but not ER-negative breast cancer liability is that the effect is in part mediated by the downstream conversion to oestradiol [42]. A negative association between SHBG levels and breast cancer was observed in a meta-analysis of prospective studies [44], and in an MR study, only after adjusting for BMI in an MVMR model [30], in agreement with our study where we only found an association after adjusting for BMI for ER-positive breast cancer liability.

For cardiovascular biomarkers, a cohort study found an inverse association between ApoB, but not ApoA and breast cancer risk [45]. However, our MR study found a positive association between genetically predicted ApoA, but not genetically predicted ApoB and overall breast cancer liability. This difference in findings may have arisen due to confounding or reverse causation in the prospective cohort study. A meta-analysis of 15 observational studies did not find evidence of an association between CRP levels and overall breast cancer liability [46], in agreement with a previous MR study [47] and the current MR study. No evidence of associations of cholesterol, HDL cholesterol, LDL cholesterol, and triglycerides with overall breast cancer risk was found in a meta-analysis of cohort studies [48]. An MR study also found no evidence of associations of genetically predicted cholesterol, LDL cholesterol, or triglycerides, but found a positive association of HDL cholesterol with overall breast cancer [49]. We also found no evidence of associations of genetically predicted cholesterol or LDL cholesterol with overall breast cancer. However, we observed that genetically raised HDL cholesterol was consistently significantly positively associated with all breast cancer outcomes. HDL cholesterol has been shown to stimulate breast cancer cell line proliferation in a dose-dependent relationship. The HDL receptor scavenger receptor class B type I, which contributes to tumour development via AKT and ERK1/2, has also been shown to be expressed more abundantly in human breast cancer tissue than in non-cancerous tissue [50]. Triglycerides were associated with a decreased liability for breast cancer, although not significantly in MVMR including the other lipids, and not ranked highly in MR-BMA. There was no evidence of an association between genetically predicted lipoprotein A levels and breast cancer liability in our study.

For diabetes-related biomarkers, a meta-analysis of 10 cohort studies [51] and a previous MR study [52] found evidence of a positive association between serum glucose levels and risk or odds of overall breast cancer. However, we did not observe any evidence of association. We did not observe any associations between genetically predicted glycated haemoglobin levels and breast cancer liability.

For liver biomarkers, a case-cohort study found an inverse association between albumin and breast cancer risk [53], while our MR analyses did not find any evidence of association, likely due to residual confounding in the case-cohort study. The results of a meta-analysis of two cohort studies showed a higher risk of breast cancer with higher gamma-glutamyltransferase concentrations [54], whereas we did not find any evidence of an association in our MR study, possibly due to confounding in the cohort studies. No evidence of an association between total bilirubin concentrations and overall breast cancer liability was found in a case-cohort study [53], in agreement with our MR results. We found evidence of an inverse association between genetically predicted aspartate aminotransferase concentrations and overall or ER-positive breast cancer liability. Yet, given the inconclusive evidence from our pleiotropy-robust approaches, possible bias from pleiotropy could not be excluded. We did not find any evidence of associations between genetically predicted alanine aminotransferase or direct bilirubin levels with breast cancer liability.

For renal biomarkers, a case-cohort study found an inverse association between uric acid levels and overall breast cancer liability. However, following adjustment for albumin, the association was attenuated [53]. Our study found no evidence of an association between genetically predicted urate levels and breast cancer liability. We found evidence for an inverse association between genetically predicted urea levels and ER-positive breast cancer liability, but the evidence was inconclusive in the pleiotropy-robust approaches due to large uncertainties that included the null, meaning that our results were more suggestive of a lack of association. We could not find any evidence of associations of genetically predicted serum creatinine, enzymatic creatinine, cystatin C, microalbumin, phosphate, potassium, sodium, and total protein with breast cancer liability.

A limitation of our study was that the data was restricted to women of white-European ancestry to avoid heterogeneity issues, which hinders our ability to generalise to populations of other ethnic backgrounds. Another deficit of our study was that our exposure [12] and outcome [8] samples were predominantly post-menopausal, thus limiting generalisability to pre-menopausal women. Moreover, though we performed multiple MR sensitivity analyses, there is still the possibility of residual pleiotropy.

Our study’s strengths include applying many univariable sensitivity analyses to appraise the validity of IV assumptions and limit potential bias from pleiotropy. We also included several MVMR models in our study to adjust for potential risk factors. To investigate reverse causation, we also conducted bidirectional MR for the association between genetically predicted ALP concentrations and breast cancer liability. Biomarker samples were collected prospectively from a large sample, and we accounted for population stratification by restricting our study to participants of white-European ethnicity and adjusting for genetic principal components. We explored genetic associations in women, which excluded the potential for sex-specific effects that can arise for biomarkers such as testosterone [55]. Most of our results supported findings from previous studies, which acted as positive controls for our methods. Our study allowed for the generation of hypotheses, enabling further studies to be targeted at biomarkers of interest with little prior evidence of association, such as ALP. Ranking biomarkers in an agnostic manner using MR-BMA reinforced our confidence in the strength of our findings and provided us with information about the importance of testosterone, HDL cholesterol, IGF-1, and ALP in breast cancer liability.

## Conclusions

We performed the most comprehensive and largest exploratory MR study to investigate the associations between all UKB biomarkers and overall, ER-positive, and ER-negative breast cancer. We replicated previous findings by corroborating the breast cancer liability-increasing effects of testosterone, HDL cholesterol, and IGF-1 and generated the novel hypothesis that ALP is potentially liability-decreasing. Further research into the association between ALP and breast cancer liability is required, for example through an MVMR adjusting for oestrogen, to understand its mechanism in breast cancer risk.

## Supplementary Information

**Additional file 1: Supplementary Tables. Table S1.** In the literature, the risk and odds of overall breast cancer per unit increase in biomarker level. Summary table of the most recent and largest studies on the relationship between each UKB biomarker and overall breast cancer. A unit is defined differently in each study. Results in bold font are significant. A dash (-) indicates that no study could be identified in the literature. BC, total breast cancer; PHR, pooled hazards ratio; PRR, pooled risk ratio; POR, pooled odds ratio, SRR, summary risk ratio; preM, pre-menopause; postM, post-menopause; IVW MR, inverse-variance weighted Mendelian randomisation. **Table S2.** Results from bidirectional MR analyses of the effects of genetically predicted overall breast cancer liability on genetically predicted alkaline phosphatase levels. Int. P-value; intercept P-value of MR-Egger. **Table S3.** Ranking of individual biomarkers according to their MIP with a PP of inclusion of 0.25 for overall breast cancer. MR-BMA ranking of the seven biomarkers nominally significantly associated with overall breast cancer in IVW MR and with consistent effect directions in the sensitivity analyses. **Table S4.** An MR-BMA ranking of individual biomarkers according to their marginal inclusion probability with a prior probability of inclusion of 0.75 for overall breast cancer. A ranking of the seven biomarkers nominally significantly associated with overall breast cancer in IVW MR and with consistent effect directions in the sensitivity analyses. **Table S5.** An MR-BMA ranking of the top 20 models according to their posterior probability with a prior probability of inclusion of 0.5 for overall breast cancer liability. Models consist of different combinations of the seven biomarkers nominally significantly associated with overall breast cancer in IVW MR and with consistent effect directions in the sensitivity analyses. **Table S6.** An MR-BMA ranking of the top 20 models according to their posterior probability with a prior probability of inclusion of 0.25 for overall breast cancer liability. Models consist of different combinations of the seven biomarkers nominally significantly associated with overall breast cancer in IVW MR and with consistent effect directions in the sensitivity analyses. **Table S7.** An MR-BMA ranking of the top 20 models according to their posterior probability with a prior probability of inclusion of 0.75 for overall breast cancer liability. Models consist of different combinations of the seven biomarkers nominally significantly associated with overall breast cancer in IVW MR and with consistent effect directions in the sensitivity analyses. **Table S8.** Results from bidirectional MR analyses of the effects of genetically predicted ER-positive breast cancer liability on genetically predicted alkaline phosphatase levels. Int. P-value; intercept P-value of MR-Egger. **Table S9.** Results from bidirectional MR analyses of the effects of genetically predicted ER-negative breast cancer liability on genetically predicted alkaline phosphatase levels. Int. P-value; intercept P-value of MR-Egger.

**Additional file 2:**  STROBE-MR Checklist.

**Additional file 3: **SNP Information. **Table 1.** Secondary trait associations of HDL cholesterol SNPs. Phenoscanner SNP associations. SNP: single nucleotide polymorphism. hg19_coordinates: the hg19 chromosome position for the input SNP. hg38_coordinates: the hg38 chromosome position for the input SNP. a1: the effect allele (aligned to the + strand). a2: the non-effect allele (aligned to the + strand). efo: the EFO ontology term for the phenotype or disease. pmid: PubMed ID. beta: association between the trait and the SNP expressed per additional copy of the effect allele (odds ratios are given on the log-scale). se: standard error of beta. p: p-value. direction: the direction of association with respect to the effect allele. n: number of individuals. n_cases: number of cases. n_controls: number of controls. n_studies: number of studies. unit: unit of analysis (IVNT stands for inverse normally rank transformed phenotype). **Table 2.** Secondary trait associations of alkaline phosphatase SNPs. Phenoscanner SNP associations. SNP: single nucleotide polymorphism. hg19_coordinates: the hg19 chromosome position for the input SNP. hg38_coordinates: the hg38 chromosome position for the input SNP. a1: the effect allele (aligned to the + strand). a2: the non-effect allele (aligned to the + strand). efo: the EFO ontology term for the phenotype or disease. pmid: PubMed ID. beta: association between the trait and the SNP expressed per additional copy of the effect allele (odds ratios are given on the log-scale). se: standard error of beta. p: p-value. direction: the direction of association with respect to the effect allele. n: number of individuals. n_cases: number of cases. n_controls: number of controls. n_studies: number of studies. unit: unit of analysis (IVNT stands for inverse normally rank transformed phenotype). **Table 3.** Secondary trait associations of testosterone SNPs. Phenoscanner SNP associations. SNP: single nucleotide polymorphism. hg19_coordinates: the hg19 chromosome position for the input SNP. hg38_coordinates: the hg38 chromosome position for the input SNP. a1: the effect allele (aligned to the + strand). a2: the non-effect allele (aligned to the + strand). efo: the EFO ontology term for the phenotype or disease. pmid: PubMed ID. beta: association between the trait and the SNP expressed per additional copy of the effect allele (odds ratios are given on the log-scale). se: standard error of beta. p: p-value. direction: the direction of association with respect to the effect allele. n: number of individuals. n_cases: number of cases. n_controls: number of controls. n_studies: number of studies. unit: unit of analysis (IVNT stands for inverse normally rank transformed phenotype). **Table 4.** Secondary trait associations of triglycerides SNPs. Phenoscanner SNP associations. SNP: single nucleotide polymorphism. hg19_coordinates: the hg19 chromosome position for the input SNP. hg38_coordinates: the hg38 chromosome position for the input SNP. a1: the effect allele (aligned to the + strand). a2: the non-effect allele (aligned to the + strand). efo: the EFO ontology term for the phenotype or disease. pmid: PubMed ID. beta: association between the trait and the SNP expressed per additional copy of the effect allele (odds ratios are given on the log-scale). se: standard error of beta. p: p-value. direction: the direction of association with respect to the effect allele. n: number of individuals. n_cases: number of cases. n_controls: number of controls. n_studies: number of studies. unit: unit of analysis (IVNT stands for inverse normally rank transformed phenotype). **Table 5.** Secondary trait associations of IGF-1 SNPs. Phenoscanner SNP associations. SNP: single nucleotide polymorphism. hg19_coordinates: the hg19 chromosome position for the input SNP. hg38_coordinates: the hg38 chromosome position for the input SNP. a1: the effect allele (aligned to the + strand). a2: the non-effect allele (aligned to the + strand). efo: the EFO ontology term for the phenotype or disease. pmid: PubMed ID. beta: association between the trait and the SNP expressed per additional copy of the effect allele (odds ratios are given on the log-scale). se: standard error of beta. p: p-value. direction: the direction of association with respect to the effect allele. n: number of individuals. n_cases: number of cases. n_controls: number of controls. n_studies: number of studies. unit: unit of analysis (IVNT stands for inverse normally rank transformed phenotype). **Table 6.** Secondary trait associations of apolipoprotein A SNPs. Phenoscanner SNP associations. SNP: single nucleotide polymorphism. hg19_coordinates: the hg19 chromosome position for the input SNP. hg38_coordinates: the hg38 chromosome position for the input SNP. a1: the effect allele (aligned to the + strand). a2: the non-effect allele (aligned to the + strand). efo: the EFO ontology term for the phenotype or disease. pmid: PubMed ID. beta: association between the trait and the SNP expressed per additional copy of the effect allele (odds ratios are given on the log-scale). se: standard error of beta. p: p-value. direction: the direction of association with respect to the effect allele. n: number of individuals. n_cases: number of cases. n_controls: number of controls. n_studies: number of studies. unit: unit of analysis (IVNT stands for inverse normally rank transformed phenotype). **Table 7.** Secondary trait associations of aspartate aminotransferase SNPs. Phenoscanner SNP associations. SNP: single nucleotide polymorphism. hg19_coordinates: the hg19 chromosome position for the input SNP. hg38_coordinates: the hg38 chromosome position for the input SNP. a1: the effect allele (aligned to the + strand). a2: the non-effect allele (aligned to the + strand). efo: the EFO ontology term for the phenotype or disease. pmid: PubMed ID. beta: association between the trait and the SNP expressed per additional copy of the effect allele (odds ratios are given on the log-scale). se: standard error of beta. p: p-value. direction: the direction of association with respect to the effect allele. n: number of individuals. n_cases: number of cases. n_controls: number of controls. n_studies: number of studies. unit: unit of analysis (IVNT stands for inverse normally rank transformed phenotype). **Table 8.** Genetic associations with HDL cholesterol, overall , ER-positive, and ER-negative breast cancers. Abbreviations: SNP, single nucleotide polymorphism; Alt, alternate allele (not necessarily minor allele); Ref, reference allele; SE, standard error; P, P-value; MAF, minor allele frequency (equal to ref allele when AF > 0.5, otherwise equal to alt allele - calculated using hardcall genotypes); OBC, overall breast cancer; ERpos BC, ER-positive breast cancer; ERneg BC, ER-negative breast cancer. **Table 9.** Genetic associations with alkaline phosphatase, overall, ER-positive, and ER-negative breast cancers. Abbreviations: SNP, single nucleotide polymorphism; Alt, alternate allele (not necessarily minor allele); Ref, reference allele; SE, standard error; P, P-value; MAF, minor allele frequency (equal to ref allele when AF > 0.5, otherwise equal to alt allele - calculated using hardcall genotypes); OBC, overall breast cancer; ERpos BC, ER positive breast cancer; ERneg BC, ER negative breast cancer. **Table 10.** Genetic associations with testosterone, overall , ER-positive, and ER-negative breast cancers. Abbreviations: SNP, single nucleotide polymorphism; Alt, alternate allele (not necessarily minor allele); Ref, reference allele; SE, standard error; P, P-value; MAF, minor allele frequency (equal to ref allele when AF > 0.5, otherwise equal to alt allele - calculated using hardcall genotypes); OBC, overall breast cancer; ERpos BC, ER positive breast cancer; ERneg BC, ER negative breast cancer. **Table 11.** Genetic associations with triglycerides, overall , ER-positive, and ER-negative breast cancers. Abbreviations: SNP, single nucleotide polymorphism; Alt, alternate allele (not necessarily minor allele); Ref, reference allele; SE, standard error; P, P-value; MAF, minor allele frequency (equal to ref allele when AF > 0.5, otherwise equal to alt allele - calculated using hardcall genotypes); OBC, overall breast cancer; ERpos BC, ER positive breast cancer; ERneg BC, ER negative breast cancer. **Table 12.** Genetic associations with IGF-1, overall , ER-positive, and ER-negative breast cancers. Abbreviations: SNP, single nucleotide polymorphism; Alt, alternate allele (not necessarily minor allele); Ref, reference allele; SE, standard error; P, P-value; MAF, minor allele frequency (equal to ref allele when AF > 0.5, otherwise equal to alt allele - calculated using hardcall genotypes); OBC, overall breast cancer; ERpos BC, ER positive breast cancer; ERneg BC, ER negative breast cancer. **Table 13.** Genetic associations with apolipoprotein A, overall , ER-positive, and ER-negative breast cancers. Abbreviations: SNP, single nucleotide polymorphism; Alt, alternate allele (not necessarily minor allele); Ref, reference allele; SE, standard error; P, P-value; MAF, minor allele frequency (equal to ref allele when AF > 0.5, otherwise equal to alt allele - calculated using hardcall genotypes); OBC, overall breast cancer; ERpos BC, ER positive breast cancer; ERneg BC, ER negative breast cancer. **Table 14.** Genetic associations with aspartate aminotransferase, overall , ER-positive, and ER-negative breast cancers. Abbreviations: SNP, single nucleotide polymorphism; Alt, alternate allele (not necessarily minor allele); Ref, reference allele; SE, standard error; P, P-value; MAF, minor allele frequency (equal to ref allele when AF > 0.5, otherwise equal to alt allele - calculated using hardcall genotypes); OBC, overall breast cancer; ERpos BC, ER-positive breast cancer; ERneg BC, ER-negative breast cancer.

**Additional file 4: Supplementary Figures. Figure S1.** MR forest plot of bone and joint biomarkers on overall breast cancer liability. The forest plot in the centre displays the odds ratio of the effect of a SD increase in genetically predicted biomarker concentration on overall breast cancer liability as a square, with error bars representing the 95% CI. In addition to the main analysis based on IVW MR, we include sensitivity analyses based on the weighted median, MR-Egger, MR-PRESSO, and MVMR accounting for known pleiotropic pathways. N. SNPs; number of SNPs. CI; confidence interval. Int. P-value; intercept P-value of MR-Egger. T; Testosterone. BMI; body mass index. An asterisk (*) indicates nominal significance. Two asterisks (**) indicate FDR corrected significance. **Figure S2.** MR forest plot of cancer biomarkers on overall breast cancer liability. The forest plot in the centre displays the odds ratio of the effect of a SD increase in genetically predicted biomarker concentration on overall breast cancer liability as a square, with error bars representing the 95% CI. In addition to the main analysis based on IVW MR, we include sensitivity analyses based on the weighted median, MR-Egger, MR-PRESSO, and MVMR accounting for known pleiotropic pathways. N. SNPs; number of SNPs. Int. P-value; intercept P-value. ALP; alkaline phosphatase. T; testosterone. BMI; body mass index. * indicates nominal significance. An asterisk (*) indicates nominal significance. Two asterisks (**) indicate FDR corrected significance. **Figure S3.** MR forest plot of cardiovascular biomarkers on overall breast cancer liability. The forest plot in the centre displays the odds ratio of the effect of a SD increase in genetically predicted biomarker concentration on overall breast cancer liability as a square, with error bars representing the 95% CI. In addition to the main analysis based on IVW MR, we include sensitivity analyses based on the weighted median, MR-Egger, MR-PRESSO, and MVMR accounting for known pleiotropic pathways. N. SNPs; number of SNPs. Int. P-value; intercept P-value. BMI; body mass index. An asterisk (*) indicates nominal significance. Two asterisks (**) indicate FDR corrected significance. **Figure S4.** MR forest plot of diabetes biomarkers on overall breast cancer liability. The forest plot in the centre displays the odds ratio of the effect of a SD increase in genetically predicted biomarker concentration on overall breast cancer liability as a square, with error bars representing the 95% CI. In addition to the main analysis based on IVW MR, we include sensitivity analyses based on the weighted median, MR-Egger, MR-PRESSO, and MVMR accounting for known pleiotropic pathways. N. SNPs; number of SNPs. Int. P-value; intercept P-value. An asterisk (*) indicates nominal significance. Two asterisks (**) indicate FDR corrected significance. **Figure S5.** MR forest plot of liver biomarkers on overall breast cancer liability. The forest plot in the centre displays the odds ratio of the effect of a SD increase in genetically predicted biomarker concentration on overall breast cancer liability as a square, with error bars representing the 95% CI. In addition to the main analysis based on IVW MR, we include sensitivity analyses based on the weighted median, MR-Egger, MR-PRESSO, and MVMR accounting for known pleiotropic pathways. N. SNPs; number of SNPs. Int. P-value; intercept P-value. An asterisk (*) indicates nominal significance. Two asterisks (**) indicate FDR corrected significance. **Figure S6.** MR forest plot of renal biomarkers on overall breast cancer liability. The forest plot in the centre displays the odds ratio of the effect of a SD increase in genetically predicted biomarker concentration on overall breast cancer liability as a square, with error bars representing the 95% CI. In addition to the main analysis based on IVW MR, we include sensitivity analyses based on the weighted median, MR-Egger, MR-PRESSO, and MVMR accounting for known pleiotropic pathways. N. SNPs; number of SNPs. Int. P-value; intercept P-value. An asterisk (*) indicates nominal significance. Two asterisks (**) indicate FDR corrected significance. **Figure S7.** MVMR forest plot of lipid biomarkers on overall breast cancer liability. The forest plot displays the odds ratio of the effect of a unit increase in genetically predicted biomarker concentration on overall breast cancer liability as a square, with error bars representing the 95% CI. Biomarkers are shown in descending order of significance. An asterisk (*) indicates nominal significance. **Figure S8.** MVMR forest plot of biomarkers, alcohol, and BMI on overall breast cancer liability. The forest plot displays the odds ratio of the effect of a unit increase in genetically predicted biomarker concentration on overall breast cancer liability as a square, with error bars representing the 95% CI. Biomarkers are shown in descending order of significance. An asterisk (*) indicates nominal significance. **Figure S9.** MVMR forest plot of sex hormone biomarkers on overall breast cancer liability. The forest plot displays the odds ratio of the effect of a unit increase in genetically predicted biomarker concentration on overall breast cancer liability as a square, with error bars representing the 95% CI. Biomarkers are shown in descending order of significance. An asterisk (*) indicates nominal significance. **Figure S10.** MVMR forest plot of BMI and SHBG on overall breast cancer liability. The forest plot displays the odds ratio of the effect of a unit increase in genetically predicted biomarker concentration on overall breast cancer liability as a square, with error bars representing the 95% CI. Biomarkers are shown in descending order of significance. An asterisk (*) indicates nominal significance. **Figure S11.** MR forest plot of cardiovascular biomarkers on ER-positive breast cancer liability. The forest plot in the centre displays the odds ratio of the effect of a SD increase in genetically predicted biomarker concentration on ER positive breast cancer liability as a square, with error bars representing the 95% CI. In addition to the main analysis based on IVW MR, we include sensitivity analyses based on the weighted median, MR-Egger, MR-PRESSO, and MVMR accounting for known pleiotropic pathways. N. SNPs; number of SNPs. Int. P-value; intercept P-value. BMI; body mass index. An asterisk (*) indicates nominal significance. Two asterisks (**) indicate FDR corrected significance. **Figure S12.** MR forest plot of bone and joint biomarkers on ER-positive breast cancer liability. The forest plot in the centre displays the odds ratio of the effect of a SD increase in genetically predicted biomarker concentration on ER positive breast cancer liability as a square, with error bars representing the 95% CI. In addition to the main analysis based on IVW MR, we include sensitivity analyses based on the weighted median, MR-Egger, MR-PRESSO, and MVMR accounting for known pleiotropic pathways. N. SNPs; number of SNPs. Int. P-value; intercept P-value. BMI; body mass index. An asterisk (*) indicates nominal significance. Two asterisks (**) indicate FDR corrected significance. **Figure S13.** MR forest plot of cancer biomarkers on ER-positive breast cancer liability. The forest plot in the centre displays the odds ratio of the effect of a SD increase in genetically predicted biomarker concentration on ER negative breast cancer liability as a square, with error bars representing the 95% CI. In addition to the main analysis based on IVW MR, we include sensitivity analyses based on the weighted median, MR-Egger, MR-PRESSO, and MVMR accounting for known pleiotropic pathways. N. SNPs; number of SNPs. Int. P-value; intercept P-value. BMI; body mass index. T; testosterone. An asterisk (*) indicates nominal significance. Two asterisks (**) indicate FDR corrected significance. **Figure S14.** MR forest plot of diabetes biomarkers on ER-positive breast cancer liability. The forest plot in the centre displays the odds ratio of the effect of a SD increase in genetically predicted biomarker concentration on ER negative breast cancer liability as a square, with error bars representing the 95% CI. In addition to the main analysis based on IVW MR, we include sensitivity analyses based on the weighted median, MR-Egger, MR-PRESSO, and MVMR accounting for known pleiotropic pathways. N. SNPs; number of SNPs. Int. P-value; intercept P-value. BMI; body mass index. T; testosterone. ALP; alkaline phosphatase. An asterisk (*) indicates nominal significance. Two asterisks (**) indicate FDR corrected significance. **Figure S15.** MR forest plot of liver biomarkers on ER-positive breast cancer liability. The forest plot in the centre displays the odds ratio of the effect of a SD increase in genetically predicted biomarker concentration on ER negative breast cancer liability as a square, with error bars representing the 95% CI. In addition to the main analysis based on IVW MR, we include sensitivity analyses based on the weighted median, MR-Egger, MR-PRESSO, and MVMR accounting for known pleiotropic pathways. N. SNPs; number of SNPs. Int. P-value; intercept P-value. BMI; body mass index. An asterisk (*) indicates nominal significance. Two asterisks (**) indicate FDR corrected significance. **Figure S16.** MR forest plot of renal biomarkers on ER-positive breast cancer liability. The forest plot in the centre displays the odds ratio of the effect of a SD increase in genetically predicted biomarker concentration on ER negative breast cancer liability as a square, with error bars representing the 95% CI. In addition to the main analysis based on IVW MR, we include sensitivity analyses based on the weighted median, MR-Egger, MR-PRESSO, and MVMR accounting for known pleiotropic pathways. N. SNPs; number of SNPs. Int. P-value; intercept P-value. An asterisk (*) indicates nominal significance. Two asterisks (**) indicate FDR corrected significance. **Figure S17.** MR forest plot of bone and joint biomarkers on ER-negative breast cancer liability. The forest plot in the centre displays the odds ratio of the effect of a SD increase in genetically predicted biomarker concentration on ER negative breast cancer liability as a square, with error bars representing the 95% CI. In addition to the main analysis based on IVW MR, we include sensitivity analyses based on the weighted median, MR-Egger, MR-PRESSO, and MVMR accounting for known pleiotropic pathways. N. SNPs; number of SNPs. Int. P-value; intercept P-value. An asterisk (*) indicates nominal significance. Two asterisks (**) indicate FDR corrected significance. **Figure S18.** MR forest plot of cancer biomarkers on ER-negative breast cancer liability. The forest plot in the centre displays the odds ratio of the effect of a SD increase in genetically predicted biomarker concentration on ER negative breast cancer liability as a square, with error bars representing the 95% CI. In addition to the main analysis based on IVW MR, we include sensitivity analyses based on the weighted median, MR-Egger, MR-PRESSO, and MVMR accounting for known pleiotropic pathways. N. SNPs; number of SNPs. Int. P-value; intercept P-value. An asterisk (*) indicates nominal significance. Two asterisks (**) indicate FDR corrected significance. **Figure S19.** MR forest plot of cardiovascular biomarkers on ER-negative breast cancer liability. The forest plot displays the odds ratio of the effect of a unit increase in genetically predicted biomarker concentration on overall breast cancer liability as a square, with error bars representing the 95% CI. Biomarkers are shown in descending order of significance. An asterisk (*) indicates nominal significance. **Figure S20.** MR forest plot of diabetes biomarkers on ER-negative breast cancer liability. The forest plot displays the odds ratio of the effect of a unit increase in genetically predicted biomarker concentration on overall breast cancer liability as a square, with error bars representing the 95% CI. Biomarkers are shown in descending order of significance. An asterisk (*) indicates nominal significance. **Figure S21.** MR forest plot of liver biomarkers on ER-negative breast cancer liability. The forest plot displays the odds ratio of the effect of a unit increase in genetically predicted biomarker concentration on overall breast cancer liability as a square, with error bars representing the 95% CI. Biomarkers are shown in descending order of significance. An asterisk (*) indicates nominal significance. **Figure S22.** MR forest plot of renal biomarkers on ER-negative breast cancer liability. The forest plot displays the odds ratio of the effect of a unit increase in genetically predicted biomarker concentration on overall breast cancer liability as a square, with error bars representing the 95% CI. Biomarkers are shown in descending order of significance. An asterisk (*) indicates nominal significance. **Figure S23.** MVMR forest plot of lipid biomarkers on ER-positive breast cancer liability. The forest plot displays the odds ratio of the effect of a unit increase in genetically predicted biomarker concentration on ER positive breast cancer liability as a square, with error bars representing the 95% CI. Biomarkers are shown in descending order of significance. An asterisk (*) indicates nominal significance. **Figure S24.** MVMR forest plot of lipid biomarkers on ER-negative breast cancer liability. The forest plot displays the odds ratio of the effect of a unit increase in genetically predicted biomarker concentration on ER-negative breast cancer liability as a square, with error bars representing the 95% CI. Biomarkers are shown in descending order of significance. An asterisk (*) indicates nominal significance. **Figure S25.** MVMR forest plot of biomarkers, alcohol, and BMI on ER-positive breast cancer liability. The forest plot displays the odds ratio of the effect of a unit increase in genetically predicted biomarker concentration on ER-negative breast cancer liability as a square, with error bars representing the 95% CI. Biomarkers are shown in descending order of significance. An asterisk (*) indicates nominal significance. **Figure S26.** MVMR forest plot of biomarkers, alcohol, and BMI on ER-negative breast cancer liability. The forest plot displays the odds ratio of the effect of a unit increase in genetically predicted biomarker concentration on ER positive breast cancer liability as a square, with error bars representing the 95% CI. Biomarkers are shown in descending order of significance. An asterisk (*) indicates nominal significance. **Figure S27.** MVMR forest plot of T, SHBG, and ALP on ER-positive breast cancer liability. The forest plot displays the odds ratio of the effect of a unit increase in genetically predicted biomarker concentration on ER-positive breast cancer liability as a square, with error bars representing the 95% CI. Biomarkers are shown in descending order of significance. An asterisk (*) indicates nominal significance. **Figure S28.** MVMR forest plot of lipid biomarkers on ER-negative breast cancer liability. The forest plot displays the odds ratio of the effect of a unit increase in genetically predicted biomarker concentration on ER-negative breast cancer liability as a square, with error bars representing the 95% CI. Biomarkers are shown in descending order of significance. An asterisk (*) indicates nominal significance. **Figure S29.** MVMR forest plot of BMI and SHBG on ER-positive breast cancer liability. The forest plot displays the odds ratio of the effect of a unit increase in genetically predicted biomarker concentration on ER-positive breast cancer liability as a square, with error bars representing the 95% CI. Biomarkers are shown in descending order of significance. An asterisk (*) indicates nominal significance. **Figure S30.** MVMR forest plot of BMI and SHBG on ER-negative breast cancer liability. The forest plot displays the odds ratio of the effect of a unit increase in genetically predicted biomarker concentration on ER-negative breast cancer liability as a square, with error bars representing the 95% CI. Biomarkers are shown in descending order of significance. An asterisk (*) indicates nominal significance.

**Additional file 5: Table S1.** Risk and odds of breast cancer per unit increase of each UKB biomarker in the literature and our study. A unit is defined differently in each study. Results in bold font are significant. BC, total breast cancer; PHR, pooled hazards ratio; PRR, pooled risk ratio; POR, pooled odds ratio, SRR, summary risk ratio; preM, pre-menopause; postM, post-menopause; IVW MR, inverse-variance weighted Mendelian randomisation. An asterisk (*) indicates that the ratio method was performed.

## Data Availability

We thank the participants and researchers for making the summary-level data used in this study publicly available. The summary-level GWAS statistics for the exposures were obtained from Neale et al. [9]. The GWAS summary statistics of overall breast cancer cases and controls were obtained from the BCAC [13].

## Abbreviations

ALP: Alkaline phosphatase
Apo: Apolipoprotein
BCAC: Breast Cancer Association Consortium
BMI: Body mass index
CI: Confidence interval
CRP: C-reactive protein
ER: Oestrogen receptor
HDL: High-density lipoprotein
IGF-1: Insulin-like growth factor 1
IV: Instrumental variable
MIP: Marginal inclusion probability
MR: Mendelian randomisation
MR-BMA: MR Bayesian model averaging
MR-PRESSO: MR Pleiotropy RESidual Sum and Outlier
MVMR: Multivariable MR
OR: Odds ratio
PP: Posterior probability
PRR: Population risk ratio
RR: Risk ratio
SD: Standard deviation
SRR: Summary relative risk
UKB: UK Biobank
