# Supplementary material for: Identifying and ranking causal biochemical biomarkers for breast cancer: a Mendelian randomisation study
Source: BMC Med. 2022 Nov 23;20:457. doi: 10.1186/s12916-022-02660-2 (PMC9685978; doi:10.1186/s12916-022-02660-2)
Supplement: Supplementary file 1 — Additional file 1: Supplementary Tables. Table S1. In the literature, the risk and odds of overall breast cancer per unit increase in biomarker level. Summary table of the most recent and largest studies on the relationship between each UKB biomarker and overall breast cancer. A unit is defined differently in each study. Results in bold font are significant. A dash (-) indicates that no study could be identified in the literature. BC, total breast cancer; PHR, pooled hazards ratio; PRR, pooled risk ratio; POR, pooled odds ratio, SRR, summary risk ratio; preM, pre-menopause; postM, post-menopause; IVW MR, inverse-variance weighted Mendelian randomisation. Table S2. Results from bidirectional MR analyses of the effects of genetically predicted overall breast cancer liability on genetically predicted alkaline phosphatase levels. Int. P-value; intercept P-value of MR-Egger. Table S3. Ranking of individual biomarkers according to their MIP with a PP of inclusion of 0.25 for overall breast cancer. MR-BMA ranking of the seven biomarkers nominally significantly associated with overall breast cancer in IVW MR and with consistent effect directions in the sensitivity analyses. Table S4. An MR-BMA ranking of individual biomarkers according to their marginal inclusion probability with a prior probability of inclusion of 0.75 for overall breast cancer. A ranking of the seven biomarkers nominally significantly associated with overall breast cancer in IVW MR and with consistent effect directions in the sensitivity analyses. Table S5. An MR-BMA ranking of the top 20 models according to their posterior probability with a prior probability of inclusion of 0.5 for overall breast cancer liability. Models consist of different combinations of the seven biomarkers nominally significantly associated with overall breast cancer in IVW MR and with consistent effect directions in the sensitivity analyses. Table S6. An MR-BMA ranking of the top 20 models according to their posterior probability [file 12916_2022_2660_MOESM1_ESM.docx]

**Additional file 1: Supplementary Tables.**

| **Biomarker group** | **Biomarker name** | **Risk of overall breast cancer (95% CI)** | **IVW MR odds of overall breast cancer (95% CI)** |
| --- | --- | --- | --- |
| Bone and joint | Alkaline phosphatase | - | - |
|  | Calcium | **Meta-analysis PHR: 0.80 (0.66-0.97) (Wulaningsih, 2016)** | OR: 0.91 (0.83-1.13) (Papadimitriou, 2020) |
|  | Rheumatoid factor | - | - |
|  | Vitamin D | **Meta-analysis PRR: 0.73 (0.60-0.88) (Yin, 2010)** | OR: 1.02 (0.97-1.08) (Jiang, 2019) |
| Cancer | IGF-1 | **Prospective cohort HR: 1.11 (1.07-1.16) (Murphy, 2020)** | **OR: 1.05 (1.01-1.10) (Murphy, 2020)** |
|  | Oestradiol | **Meta-analysis OR: 2.15 (1.87-2.46) (Key, 2015)** | - |
|  | SHBG | **Meta-analysis PRR: 0.64 (0.57-0.72) (He, 2015)** | **OR: 0.94 (0.90-0.98) (Dimou, 2019)** |
|  | Testosterone | **Meta-analysis OR: 2.04 (1.76-2.37) (Key, 2015)** | **OR: 1.14 (1.08-1.20) (Ruth, 2020)** |
| Cardiovascular | Apolipoprotein A | Prospective cohort HR: 1.06 (0.99-1.14) (Borgquist, 2016) | - |
|  | Apolipoprotein B | **Prospective cohort HR: 0.92 (0.86-0.99) (Borgquist, 2016)** | - |
|  | C-reactive protein | Meta-analysis POR: 1.16 (1.06-1.27) (Guo, 2015) | OR: 1.03 (0.94-1.13) (Robinson, 2020) |
|  | Cholesterol | Meta-analysis PRR: 0.96 (0.86-1.07) (Ni, 2015) | OR: 1.05 (0.99-1.11) (Beeghly-Fadiel, 2019) |
|  | HDL cholesterol | Meta-analysis PRR: 0.92 (0.73-1.16) (Ni, 2015) | **OR: 1.12 (1.08-1.16) (Beeghly-Fadiel, 2019)** |
|  | LDL direct | Meta-analysis PRR: 0.90 (0.77-1.06) (Ni, 2015) | OR: 1.00 (0.96-1.04) (Beeghly-Fadiel, 2019) |
|  | Lipoprotein A | - | - |
|  | Triglyceride | Meta-analysis PRR: 0.93 (0.86-1.00) (Ni, 2015) | OR: 0.93 (0.85-1.01) (Beeghly-Fadiel, 2019) |
| Diabetes | Glucose | Meta-analysis SRR: 1.11 (1.00-1.23) (Boyle, 2013) | **OR: 1.80 (1.30-2.49) (Shu, 2019)** |
|  | Glycated haemoglobin | - | - |
| Liver | Alanine aminotransferase | - | - |
|  | Albumin | **Case-cohort HR: 0.71 (0.51-0.99) (Kuhn, 2017)** | - |
|  | Aspartate aminotransferase | - | - |
|  | Direct bilirubin | - | - |
|  | Gamma glutamyltransferase | **Meta-analysis PRR: 1.67 (1.12-2.48) (Kunutsor, 2015)** | - |
|  | Total bilirubin | Case-cohort HR: 0.56 (0.31-1.02) (Kuhn, 2017) | - |
| Renal | Creatinine | - | - |
|  | Creatinine (enzymatic) | - | - |
|  | Cystatin C | - | - |
|  | Microalbumin | - | - |
|  | Phosphate | - | - |
|  | Potassium | - | - |
|  | Sodium | - | - |
|  | Total protein | - | - |
|  | Urate | **Case-control HR: 0.72 (0.53-0.99) (uric acid) (Kuhn, 2017)** | - |
|  | Urea | - | - |

**Table S1. In the literature, the risk and odds of overall breast cancer per unit increase in biomarker level.**

Summary table of the most recent and largest studies on the relationship between each UKB biomarker and overall breast cancer. A unit is defined differently in each study. Results in bold font are significant. A dash (-) indicates that no study could be identified in the literature. BC, total breast cancer; PHR, pooled hazards ratio; PRR, pooled risk ratio; POR, pooled odds ratio, SRR, summary risk ratio; preM, pre-menopause; postM, post-menopause; IVW MR, inverse-variance weighted Mendelian randomisation.

**Table S2. Results from bidirectional MR analyses of the effects of genetically predicted overall breast cancer liability on genetically predicted alkaline phosphatase levels.**

| Method | Estimate | Std Error | 95% LCI | 95% UCI | P-value | Int. P-value |
| --- | --- | --- | --- | --- | --- | --- |
| IVW | 0.987 | 0.026 | 0.938 | 1.038 | 0.612 |  |
| Weighted median | 1.000 | 0.008 | 0.984 | 1.016 | 0.987 |  |
| MR-Egger | 1.030 | 0.054 | 0.927 | 1.145 | 0.576 | 0.359 |

Int. P-value; intercept P-value of MR-Egger.

**Table S3. Ranking of individual biomarkers according to their MIP with a PP of inclusion of 0.25 for overall breast cancer.**

| **Ranking** | **Exposure** | **Marginal inclusion probability** | **Model-averaged causal effect** |
| --- | --- | --- | --- |
| 1 | Testosterone | 0.961 | 0.080 |
| 2 | HDL cholesterol | 0.494 | 0.021 |
| 3 | IGF-1 | 0.376 | 0.015 |
| 4 | Alkaline phosphatase | 0.349 | -0.012 |
| 5 | Apolipoprotein A | 0.094 | -0.003 |
| 6 | Triglycerides | 0.046 | -0.001 |
| 7 | Aspartate aminotransferase | 0.013 | 0 |

MR-BMA ranking of the seven biomarkers nominally significantly associated with overall breast cancer in IVW MR and with consistent effect directions in the sensitivity analyses.

**Table S4. An MR-BMA ranking of individual biomarkers according to their marginal inclusion probability with a prior probability of inclusion of 0.75 for overall breast cancer.**

| **Ranking** | **Exposure** | **Marginal inclusion probability** | **Model-averaged causal effect** |
| --- | --- | --- | --- |
| 1 | Testosterone | 0.99 | 0.078 |
| 2 | HDL cholesterol | 0.853 | 0.052 |
| 3 | IGF-1 | 0.825 | 0.032 |
| 4 | Alkaline phosphatase | 0.799 | -0.026 |
| 5 | Apolipoprotein A | 0.448 | -0.025 |
| 6 | Triglycerides | 0.108 | -0.001 |
| 7 | Aspartate aminotransferase | 0.08 | -0.001 |

A ranking of the seven biomarkers nominally significantly associated with overall breast cancer in IVW MR and with consistent effect directions in the sensitivity analyses.

**Table S5. An MR-BMA ranking of the top 20 models according to their posterior probability with a prior probability of inclusion of 0.5 for overall breast cancer liability.**

| **Ranking** | **Models (sets of risk factors)** | **Posterior probability** | **Model-specific causal estimates** |
| --- | --- | --- | --- |
| 1 | HDL cholesterol, Alkaline phosphatase, Testosterone, IGF-1 | 0.187 | 0.034, -0.032, 0.08, 0.04 |
| 2 | HDL cholesterol, Testosterone, IGF-1 | 0.147 | 0.037, 0.08, 0.039 |
| 3 | Alkaline phosphatase, Testosterone, IGF-1 | 0.09 | -0.036, 0.081, 0.039 |
| 4 | HDL cholesterol, Alkaline phosphatase, Testosterone | 0.081 | 0.034, -0.032, 0.085 |
| 5 | HDL cholesterol, Testosterone | 0.079 | 0.037, 0.085 |
| 6 | Alkaline phosphatase, Testosterone | 0.048 | -0.035, 0.086 |
| 7 | HDL cholesterol, Alkaline phosphatase, Testosterone, IGF-1, Apolipoprotein A | 0.047 | 0.088, -0.033, 0.075, 0.037, -0.063 |
| 8 | HDL cholesterol, Alkaline phosphatase, Testosterone, Apolipoprotein A | 0.041 | 0.097, -0.032, 0.079, -0.075 |
| 9 | HDL cholesterol, Testosterone, Apolipoprotein A | 0.034 | 0.099, 0.079, -0.073 |
| 10 | HDL cholesterol, Testosterone, IGF-1, Apolipoprotein A | 0.033 | 0.089, 0.075, 0.036, -0.061 |
| 11 | Testosterone, IGF-1 | 0.032 | 0.081, 0.039 |
| 12 | Testosterone | 0.022 | 0.086 |
| 13 | Alkaline phosphatase, Testosterone, IGF-1, Apolipoprotein A | 0.021 | -0.033, 0.082, 0.041, 0.028 |
| 14 | Testosterone, IGF-1, Apolipoprotein A | 0.014 | 0.082, 0.04, 0.031 |
| 15 | Alkaline phosphatase, Testosterone, Triglycerides, IGF-1 | 0.012 | -0.033, 0.075, -0.025, 0.038 |
| 16 | Alkaline phosphatase, Testosterone, Triglycerides | 0.009 | -0.033, 0.079, -0.027 |
| 17 | Testosterone, Triglycerides, IGF-1 | 0.008 | 0.074, -0.028, 0.037 |
| 18 | Testosterone, Triglycerides | 0.008 | 0.078, -0.03 |
| 19 | Alkaline phosphatase, Testosterone, Apolipoprotein A | 0.007 | -0.033, 0.088, 0.026 |
| 20 | Testosterone, Apolipoprotein A | 0.006 | 0.088, 0.029 |

Models consist of different combinations of the seven biomarkers nominally significantly associated with overall breast cancer in IVW MR and with consistent effect directions in the sensitivity analyses.

**Table S6. An MR-BMA ranking of the top 20 models according to their posterior probability with a prior probability of inclusion of 0.25 for overall breast cancer liability.**

| **Ranking** | **Models (sets of risk factors)** | **Posterior probability** | **Model-specific causal estimates** |
| --- | --- | --- | --- |
| 1 | HDL cholesterol, Testosterone | 0.185 | 0.037, 0.085 |
| 2 | Testosterone | 0.153 | 0.086 |
| 3 | HDL cholesterol, Testosterone, IGF-1 | 0.115 | 0.037, 0.08, 0.039 |
| 4 | Alkaline phosphatase, Testosterone | 0.112 | -0.035, 0.086 |
| 5 | Testosterone, IGF-1 | 0.075 | 0.081, 0.039 |
| 6 | Alkaline phosphatase, Testosterone, IGF-1 | 0.07 | -0.036, 0.081, 0.039 |
| 7 | HDL cholesterol, Alkaline phosphatase, Testosterone | 0.063 | 0.034, -0.032, 0.085 |
| 8 | HDL cholesterol, Alkaline phosphatase, Testosterone, IGF-1 | 0.049 | 0.034, -0.032, 0.08, 0.04 |
| 9 | HDL cholesterol, Testosterone, Apolipoprotein A | 0.026 | 0.099, 0.079, -0.073 |
| 10 | Testosterone, Triglycerides | 0.018 | 0.078, -0.03 |
| 11 | Testosterone, Apolipoprotein A | 0.014 | 0.088, 0.029 |
| 12 | HDL cholesterol, Alkaline phosphatase, Testosterone, Apolipoprotein A | 0.011 | 0.097, -0.032, 0.079,  -0.075 |
| 13 | Testosterone, IGF-1, Apolipoprotein A | 0.011 | 0.082, 0.04, 0.031 |
| 14 | HDL cholesterol, Testosterone, IGF-1, Apolipoprotein A | 0.008 | 0.089, 0.075, 0.036,  -0.061 |
| 15 | HDL cholesterol, IGF-1 | 0.008 | 0.038, 0.043 |
| 16 | Alkaline phosphatase, Testosterone, Triglycerides | 0.007 | -0.033, 0.079, -0.027 |
| 17 | Testosterone, Triglycerides, IGF-1 | 0.006 | 0.074, -0.028, 0.037 |
| 18 | Alkaline phosphatase, Testosterone, Apolipoprotein A | 0.006 | -0.033, 0.088, 0.026 |
| 19 | Alkaline phosphatase, Testosterone, IGF-1, Apolipoprotein A | 0.005 | -0.033, 0.082, 0.041, 0.028 |
| 20 | HDL cholesterol | 0.005 | 0.038 |

Models consist of different combinations of the seven biomarkers nominally significantly associated with overall breast cancer in IVW MR and with consistent effect directions in the sensitivity analyses.

**Table S7. An MR-BMA ranking of the top 20 models according to their posterior probability with a prior probability of inclusion of 0.75 for overall breast cancer liability.**

| **Ranking** | **Models (sets of risk factors)** | **Posterior probability** | **Model-specific causal estimates** |
| --- | --- | --- | --- |
| 1 | HDL cholesterol, Alkaline phosphatase, Testosterone, IGF-1 | 0.269 | 0.034, -0.032, 0.08, 0.04 |
| 2 | HDL cholesterol, Alkaline phosphatase, Testosterone, IGF-1, Apolipoprotein A | 0.203 | 0.088, -0.033, 0.075, 0.037, -0.063 |
| 3 | HDL cholesterol, Testosterone, IGF-1 | 0.07 | 0.037, 0.08, 0.039 |
| 4 | HDL cholesterol, Alkaline phosphatase, Testosterone, Apolipoprotein A | 0.059 | 0.097, -0.032, 0.079, -0.075 |
| 5 | HDL cholesterol, Testosterone, IGF-1, Apolipoprotein A | 0.047 | 0.089, 0.075, 0.036, -0.061 |
| 6 | Alkaline phosphatase, Testosterone, IGF-1 | 0.043 | -0.036, 0.081, 0.039 |
| 7 | HDL cholesterol, Alkaline phosphatase, Testosterone | 0.039 | 0.034, -0.032, 0.085 |
| 8 | Alkaline phosphatase, Testosterone, IGF-1, Apolipoprotein A | 0.03 | -0.033, 0.082, 0.041, 0.028 |
| 9 | HDL cholesterol, Alkaline phosphatase, Testosterone, Triglycerides, IGF-1, Apolipoprotein A | 0.022 | 0.118, -0.034, 0.076, 0.015, 0.037, -0.091 |
| 10 | HDL cholesterol, Alkaline phosphatase, Testosterone, IGF-1, Aspartate aminotransferase | 0.022 | 0.034, -0.031, 0.079, 0.039, -0.011 |
| 11 | HDL cholesterol, Alkaline phosphatase, Testosterone, Triglycerides, IGF-1 | 0.019 | 0.03, -0.032, 0.077, -0.009, 0.039 |
| 12 | Alkaline phosphatase, Testosterone, Triglycerides, IGF-1 | 0.018 | -0.033, 0.075, -0.025, 0.038 |
| 13 | HDL cholesterol, Testosterone, Apolipoprotein A | 0.016 | -0.099, 0.079, -0.073 |
| 14 | HDL cholesterol, Alkaline phosphatase, Testosterone, IGF-1, Apolipoprotein A, Aspartate aminotransferase | 0.016 | 0.086, -0.032, 0.075, 0.037, -0.062, -0.011 |
| 15 | HDL cholesterol, Testosterone | 0.013 | 0.037, 0.085 |
| 16 | HDL cholesterol, Testosterone, IGF-1, Aspartate aminotransferase | 0.008 | 0.036, 0.079, 0.039, -0.017 |
| 17 | Alkaline phosphatase, Testosterone | 0.008 | -0.035, 0.086 |
| 18 | Testosterone, IGF-1, Apolipoprotein A | 0.007 | 0.082, 0.04, 0.031 |
| 19 | HDL cholesterol, Alkaline phosphatase, Testosterone, Triglycerides, Apolipoprotein A | 0.006 | 0.129, -0.033, 0.081, 0.016, -0.104 |
| 20 | HDL cholesterol, Testosterone, Triglycerides, IGF-1 | 0.006 | 0.032, 0.077, -0.012, 0.038 |

Models consist of different combinations of the seven biomarkers nominally significantly associated with overall breast cancer in IVW MR and with consistent effect directions in the sensitivity analyses.

**Table S8. Results from bidirectional MR analyses of the effects of genetically predicted ER-positive breast cancer liability on genetically predicted alkaline phosphatase levels.**

| Method | Estimate | Std Error | 95% LCI | 95% UCI | P-value | Int. P-value |
| --- | --- | --- | --- | --- | --- | --- |
| IVW | 1.001 | 0.007 | 0.996 | 1.024 | 0.872 |  |
| Weighted median | 0.996 | 0.007 | 0.982 | 1.010 | 0.581 |  |
| MR-Egger | 1.001 | 0.013 | 0.998 | 1.004 | 0.912 | 0.972 |

Int. P-value; intercept P-value of MR-Egger.

**Table S9. Results from bidirectional MR analyses of the effects of genetically predicted ER-negative breast cancer liability on genetically predicted alkaline phosphatase levels.**

| Method | Estimate | Std Error | 95% LCI | 95% UCI | P-value | Int. P-value |
| --- | --- | --- | --- | --- | --- | --- |
| IVW | 1.007 | 0.009 | 0.989 | 1.025 | 0.413 |  |
| Weighted median | 1.008 | 0.009 | 0.990 | 1.026 | 0.393 |  |
| MR-Egger | 1.001 | 0.025 | 0.953 | 1.051 | 0.979 | 0.778 |

Int. P-value; intercept P-value of MR-Egger.
