# Supplementary material for: Identifying and ranking causal biochemical biomarkers for breast cancer: a Mendelian randomisation study
Source: BMC Med. 2022 Nov 23;20:457. doi: 10.1186/s12916-022-02660-2 (PMC9685978; doi:10.1186/s12916-022-02660-2)
Supplement: Supplementary file 2 — Additional file 2: STROBE-MR Checklist. [file 12916_2022_2660_MOESM2_ESM.docx]

**Additional file 2: STROBE-MR Checklist.**

Checklist of recommended items to address in reports of Mendelian randomization studies^1^ ^2^.

| **Item No.** | **Section** | **Checklist item** | **Page No.** | **Relevant text from manuscript** |
| --- | --- | --- | --- | --- |
| 1 | **TITLE and ABSTRACT** | Indicate Mendelian randomization (MR) as the study’s design in the title and/or the abstract if that is a main purpose of the study | 1 | Identifying and ranking causal biochemical biomarkers for breast cancer: a Mendelian randomisation study |
|  | **INTRODUCTION** |  |  |  |
| 2 | **Background** | Explain the scientific background and rationale for the reported study. What is the exposure? Is a potential causal relationship between exposure and outcome plausible? Justify why MR is a helpful method to address the study question | 3-4 | “…the exact mechanisms of breast cancer initiation and progression are not known [2], necessitating a better understanding of disease aetiology.”  “A few observational studies have been performed to study the associations between some of the UKB biochemical biomarkers and overall breast cancer, and significant associations have been found for several biomarkers.”  “To our knowledge, fewer than half of the biochemical biomarkers in the UKB have been investigated for likely causal associations with overall breast cancer using MR…” |
| 3 | **Objectives** | State specific objectives clearly, including pre-specified causal hypotheses (if any). State that MR is a method that, under specific assumptions, intends to estimate causal effects | 4 | “This study aimed to use an MR framework to 1) explore univariable associations between genetically predicted levels of UKB biochemical biomarkers and genetic liability to overall, ER-positive, and ER-negative breast cancer, 2) investigate significant associations in detail through multivariable and bidirectional approaches, and 3) to rank the associated biomarkers by genetic evidence using a multivariable Bayesian MR approach.”  “Mendelian randomisation (MR) complements observational studies by using genetic variants as instrumental variables (IVs) to establish likely causal associations between exposures and outcomes.” |
|  | **METHODS** |  |  |  |
| 4 | **Study design and data sources** | Present key elements of the study design early in the article. Consider including a table listing sources of data for all phases of the study. For each data source contributing to the analysis, describe the following: |  |  |
|  | a) | Setting: Describe the study design and the underlying population, if possible. Describe the setting, locations, and relevant dates, including periods of recruitment, exposure, follow-up, and data collection, when available. | 5-6 | “We obtained publicly available summary-level genome-wide association study (GWAS) statistics on 34 serum, urine, and red blood cell biomarker levels, body mass index (BMI), and alcohol intake frequency from unrelated female participants of white-British ancestry (n = 194,174) in the UKB cohort study from Neale et al. [9]. The genotypes and 34 biomarker levels were collected by the UKB study at baseline between 2006 and 2010 using various laboratory techniques and instruments by different suppliers [7, 10]. The GWASes were performed using age, age^2, and the first 20 principal components (PCs) as covariates [11]. Inverse-rank normalised GWAS data was used because many of the quantitative biomarker traits were non-normally distributed. Most women (at least 59%) in the UKB cohort were post-menopausal [12]. More information about the panel of UKB biomarkers and the original UKB study can be found elsewhere [3, 7].”  “Publicly available GWAS summary statistics on overall breast cancer cases (n = 122,977) and controls (n = 105,974) of European ancestry were obtained from the BCAC [13]. Of the breast cancer cases, 69,501 were ER-positive, 21,468 were ER-negative, and the majority developed post-menopause. More details about the original studies are described elsewhere [8, 14, 15].” |
|  | b) | Participants: Give the eligibility criteria, and the sources and methods of selection of participants. Report the sample size, and whether any power or sample size calculations were carried out prior to the main analysis | 5-6 | “…unrelated female participants of white-British ancestry (n = 194,174) in the UKB cohort study...”  “Publicly available GWAS summary statistics on overall breast cancer cases (n = 122,977) and controls (n = 105,974) of European ancestry were obtained from the BCAC.” |
|  | c) | Describe measurement, quality control and selection of genetic variants | 6 | “For each exposure, we selected associated single-nucleotide polymorphisms (SNPs) at genome-wide significance (P < 5 x 10-8) and ensured their independence by removing those in linkage-disequilibrium using the PLINK method (r2 < 0.001, clumping distance = 10,000kb). We then harmonised the directions of the effect alleles between exposures and outcomes.” |
|  | d) | For each exposure, outcome, and other relevant variables, describe methods of assessment and diagnostic criteria for diseases | 6 | “Publicly available GWAS summary statistics on overall breast cancer cases (n = 122,977) and controls (n = 105,974) of European ancestry were obtained from the BCAC [13]. Of the breast cancer cases, 69,501 were ER-positive, 21,468 were ER-negative, and the majority developed post-menopause. More details about the original studies are described elsewhere [8, 14, 15].” |
|  | e) | Provide details of ethics committee approval and participant informed consent, if relevant | 29 | “The UK Biobank study obtained ethics approval from the North West Centre for Research Ethics Committee (11/NW/0382) [7]. Each study in the BCAC was approved by the ethics committee of each individual study [8].” |
| 5 | **Assumptions** | Explicitly state the three core IV assumptions for the main analysis (relevance, independence and exclusion restriction) as well assumptions for any additional or sensitivity analysis | 6 | “In all our MR analyses, SNPs must satisfy three assumptions to be considered valid IVs. Genetic variants must 1) strongly associate with the exposure (the relevance assumption), 2) be independent of confounders (the independence assumption), and 3) affect the outcome only through their effect on the exposure (the exclusion restriction assumption).” |
| 6 | **Statistical methods: main analysis** | Describe statistical methods and statistics used |  |  |
|  | a) | Describe how quantitative variables were handled in the analyses (i.e., scale, units, model) | 6 | “Inverse-rank normalised GWAS data was used because many of the quantitative biomarker traits were non-normally distributed.” |
|  | b) | Describe how genetic variants were handled in the analyses and, if applicable, how their weights were selected | 6 | “For each exposure, we selected associated single-nucleotide polymorphisms (SNPs) at genome-wide significance (P < 5 x 10-8) and ensured their independence by removing those in linkage-disequilibrium using the PLINK method (r2 < 0.001, clumping distance = 10,000kb). We then harmonised the directions of the effect alleles between exposures and outcomes.” |
|  | c) | Describe the MR estimator (e.g. two-stage least squares, Wald ratio) and related statistics. Detail the included covariates and, in case of two-sample MR, whether the same covariate set was used for adjustment in the two samples | 7 | “The main univariable analysis consisted of inverse-variance weighted (IVW) MR between each exposure and each outcome.” |
|  | d) | Explain how missing data were addressed | NA | NA |
|  | e) | If applicable, indicate how multiple testing was addressed | 7 | “We used P < 0.05 as the nominal significance threshold. We also derived false discovery rate (FDR) corrected P-values with the Benjamini-Hochberg (BH) method and used P < 0.05 as the FDR-corrected significance threshold.” |
| 7 | **Assessment of assumptions** | Describe any methods or prior knowledge used to assess the assumptions or justify their validity | 7 | “…we employed several additional univariable approaches with different underlying assumptions about the structure of the pleiotropy for all exposures, including the MR-Egger [19], weighted median [20], and MR Pleiotropy RESidual Sum and Outlier (MR-PRESSO) [21].” |
| 8 | **Sensitivity analyses and additional analyses** | Describe any sensitivity analyses or additional analyses performed (e.g. comparison of effect estimates from different approaches, independent replication, bias analytic techniques, validation of instruments, simulations) | 8 | “We performed two-sample MVMR analyses for all seven biomarkers that were nominally significantly associated with overall breast cancer in IVW MR. We searched for associations at P < 10-8 of all variants used as IVs in Phenoscanner [24, 25] (Additional file 3: T1-T7), a database providing summarised GWASes, and adjusted for traits that could be considered reasons for horizontal pleiotropy.” |
| 9 | **Software and pre-registration** |  |  |  |
|  | a) | Name statistical software and package(s), including version and settings used | 10 | “We employed the TwoSampleMR [31], MendelianRandomization [32], MRPRESSO [33], and ieugwasr [34] R packages, as well as the GitHub repository https://github.com/verena-zuber/ for MR-BMA for our analyses using R (version 4.0.5). We searched for secondary trait associations using Phenoscanner [24, 25].” |
|  | b) | State whether the study protocol and details were pre-registered (as well as when and where) | 5 | “We did not pre-register the study protocol.” |
|  | **RESULTS** |  |  |  |
| 10 | **Descriptive data** |  |  |  |
|  | a) | Report the numbers of individuals at each stage of included studies and reasons for exclusion. Consider use of a flow diagram | 5-6 | “…from unrelated female participants of white-British ancestry (n = 194,174) in the UKB cohort study…”  “Publicly available GWAS summary statistics on overall breast cancer cases (n = 122,977) and controls (n = 105,974) of European ancestry were obtained from the BCAC [13].” |
|  | b) | Report summary statistics for phenotypic exposure(s), outcome(s), and other relevant variables (e.g. means, SDs, proportions) | NA | NA |
|  | c) | If the data sources include meta-analyses of previous studies, provide the assessments of heterogeneity across these studies | NA | NA |
|  | d) | For two-sample MR:  i.  Provide justification of the similarity of the genetic variant-exposure associations between the exposure and outcome samples  ii.  Provide information on the number of individuals who overlap between the exposure and outcome studies | 5 | “Our data only includes women of European descent to reduce bias from population stratification.”  “Our study used summary-level exposure data from the UKB study [7] and summary-level outcome data from the Breast Cancer Association Consortium (BCAC) [8]. The BCAC includes ~6000 samples from the UK [8], which amounts to, at most, a ~1.4% sample overlap between the exposure and outcome samples.” |
| 11 | **Main results** |  |  |  |
|  | a) | Report the associations between genetic variant and exposure, and between genetic variant and outcome, preferably on an interpretable scale | 11 | “We used an average of 147 SNPs with F-statistics ranging from 29 to 2360 as IVs for IVW analyses for these biomarkers (Additional file 3: T8-T14).” |
|  | b) | Report MR estimates of the relationship between exposure and outcome, and the measures of uncertainty from the MR analysis, on an interpretable scale, such as odds ratio or relative risk per SD difference | 15, 20-21 | “The forest plot in the centre displays the odds ratio of the effect of an SD increase in genetically predicted concentration on overall breast cancer liability…” |
|  | c) | If relevant, consider translating estimates of relative risk into absolute risk for a meaningful time period | NA | NA |
|  | d) | Consider plots to visualize results (e.g. forest plot, scatterplot of associations between genetic variants and outcome versus between genetic variants and exposure) | Fig. 1 p. 15 and Figs. 2-3 pgs. 20-21 | “Biomarkers of nominal significance in IVW MR analyses are shown in descending order of significance. The forest plot in the centre displays the odds ratio of the effect of an SD increase in genetically predicted concentration…” |
| 12 | **Assessment of assumptions** |  |  |  |
|  | a) | Report the assessment of the validity of the assumptions | 11 | “We screened all UKB biochemical biomarkers for likely causal associations with overall breast cancer using various univariable MR methods …” |
|  | b) | Report any additional statistics (e.g., assessments of heterogeneity across genetic variants, such as *I^2^*, Q statistic or E-value) | NA | NA |
| 13 | **Sensitivity analyses and additional analyses** |  |  |  |
|  | a) | Report any sensitivity analyses to assess the robustness of the main results to violations of the assumptions | 11 | “. For these biomarkers, the weighted median, MR-Egger, and MR-PRESSO effect directions and sizes were largely consistent with our IVW MR findings, apart from the MR-Egger result for aspartate aminotransferase…” |
|  | b) | Report results from other sensitivity analyses or additional analyses | 12 | “After adjusting for BMI and alcohol in MVMR, genetically predicted HDL cholesterol, ALP, testosterone, triglycerides, IGF-1, and apoA had significant direct effects on overall breast cancer liability, while aspartate aminotransferase did not…” |
|  | c) | Report any assessment of direction of causal relationship (e.g., bidirectional MR) | 13 | “We found no evidence of an association in bidirectional MR between genetically predicted overall breast cancer liability and genetically predicted ALP concentrations…” |
|  | d) | When relevant, report and compare with estimates from non-MR analyses | 23-27 | “A nominally significant negative association between serum levels of calcium and overall breast cancer risk was found in cohort studies [36], but not in an MR study [37], with which our study concurs.” |
|  | e) | Consider additional plots to visualize results (e.g., leave-one-out analyses) | Additional file 4 |  |
|  | **DISCUSSION** |  |  |  |
| 14 | **Key results** | Summarize key results with reference to study objectives | 23 | “In this study, we used a hypothesis-generating two-sample summary-level MR approach to screen the UKB for biochemical breast cancer biomarkers. We found that increases of 1 standard deviation in the genetically predicted levels of testosterone, HDL cholesterol, IGF-1, and ALP were robustly and consistently associated with overall breast cancer liability in a variety of univariable, multivariable, bidirectional, and ranking methods based on MR. These associations remained for ER-positive breast cancer, but only HDL cholesterol remained associated with ER-negative breast cancer. To our knowledge, ALP has not been associated with breast cancer before.” |
| 15 | **Limitations** | Discuss limitations of the study, taking into account the validity of the IV assumptions, other sources of potential bias, and imprecision. Discuss both direction and magnitude of any potential bias and any efforts to address them | 27 | “A limitation of our study was that the data was restricted to women of white-European ancestry to avoid heterogeneity issues, which hinders our ability to generalise to populations of other ethnic backgrounds. Another deficit of our study was that our exposure [12] and outcome [8] samples were predominantly post-menopausal, thus limiting generalisability to pre-menopausal women. Moreover, though we performed multiple MR sensitivity analyses, there is still the possibility of residual pleiotropy.  Our study’s strengths include applying many univariable sensitivity analyses to appraise the validity of IV assumptions and limit potential bias from pleiotropy.” |
| 16 | **Interpretation** |  |  |  |
|  | a) | Meaning: Give a cautious overall interpretation of results in the context of their limitations and in comparison with other studies | 23-27 |  |
|  | b) | Mechanism: Discuss underlying biological mechanisms that could drive a potential causal relationship between the investigated exposure and the outcome, and whether the gene-environment equivalence assumption is reasonable. Use causal language carefully, clarifying that IV estimates may provide causal effects only under certain assumptions | 23-27 | “One possible explanation for this finding is that ALP-prioritized genes are enriched in primary and secondary sexual organs, and crucially, gene sets enriched among ALP-associated variants included oestradiol 17-beta-dehydrogenase activity, which catalyses oestradiol to the less potent estrone, thus reducing the risk of breast cancer [35].” |
|  | c) | Clinical relevance: Discuss whether the results have clinical or public policy relevance, and to what extent they inform effect sizes of possible interventions | NA | NA |
| 17 | **Generalizability** | Discuss the generalizability of the study results (a) to other populations, (b) across other exposure periods/timings, and (c) across other levels of exposure | 27 | “Biomarker samples were collected prospectively from a large sample, and we accounted for population stratification by restricting our study to participants of white-European ethnicity and adjusting for genetic principal components. We explored genetic associations in women, which excluded the potential for sex-specific effects that can arise for biomarkers such as testosterone [57].” |
|  | **OTHER INFORMATION** |  |  |  |
| 18 | **Funding** | Describe sources of funding and the role of funders in the present study and, if applicable, sources of funding for the databases and original study or studies on which the present study is based | 30 | “KKT was supported by Cancer Research UK (C18281/A29019).” |
| 19 | **Data and data sharing** | Provide the data used to perform all analyses or report where and how the data can be accessed, and reference these sources in the article. Provide the statistical code needed to reproduce the results in the article, or report whether the code is publicly accessible and if so, where | 29 | “We thank the participants and researchers for making the summary-level data used in this study publicly available. The summary-level GWAS statistics for the exposures were obtained from Neale et al. [9]. The GWAS summary statistics of overall breast cancer cases and controls were obtained from the BCAC [13].” |
| 20 | **Conflicts of Interest** | All authors should declare all potential conflicts of interest | 30 | “The authors declare that they have no competing interests.” |

This checklist is copyrighted by the Equator Network under the Creative Commons Attribution 3.0 Unported (CC BY 3.0) license.

1. Skrivankova VW, Richmond RC, Woolf BAR, Yarmolinsky J, Davies NM, Swanson SA, et al. Strengthening the Reporting of Observational Studies in Epidemiology using Mendelian Randomization (STROBE-MR) Statement. JAMA. 2021;under review.

2. Skrivankova VW, Richmond RC, Woolf BAR, Davies NM, Swanson SA, VanderWeele TJ, et al. Strengthening the Reporting of Observational Studies in Epidemiology using Mendelian Randomisation (STROBE-MR): Explanation and Elaboration. BMJ. 2021;375:n2233.
