# Supplementary material for: Identifying and ranking causal biochemical biomarkers for breast cancer: a Mendelian randomisation study
Source: BMC Med. 2022 Nov 23;20:457. doi: 10.1186/s12916-022-02660-2 (PMC9685978; doi:10.1186/s12916-022-02660-2)
Supplement: Supplementary file 4 — Additional file 4: Supplementary Figures. Figure S1. MR forest plot of bone and joint biomarkers on overall breast cancer liability. The forest plot in the centre displays the odds ratio of the effect of a SD increase in genetically predicted biomarker concentration on overall breast cancer liability as a square, with error bars representing the 95% CI. In addition to the main analysis based on IVW MR, we include sensitivity analyses based on the weighted median, MR-Egger, MR-PRESSO, and MVMR accounting for known pleiotropic pathways. N. SNPs; number of SNPs. CI; confidence interval. Int. P-value; intercept P-value of MR-Egger. T; Testosterone. BMI; body mass index. An asterisk (*) indicates nominal significance. Two asterisks (**) indicate FDR corrected significance. Figure S2. MR forest plot of cancer biomarkers on overall breast cancer liability. The forest plot in the centre displays the odds ratio of the effect of a SD increase in genetically predicted biomarker concentration on overall breast cancer liability as a square, with error bars representing the 95% CI. In addition to the main analysis based on IVW MR, we include sensitivity analyses based on the weighted median, MR-Egger, MR-PRESSO, and MVMR accounting for known pleiotropic pathways. N. SNPs; number of SNPs. Int. P-value; intercept P-value. ALP; alkaline phosphatase. T; testosterone. BMI; body mass index. * indicates nominal significance. An asterisk (*) indicates nominal significance. Two asterisks (**) indicate FDR corrected significance. Figure S3. MR forest plot of cardiovascular biomarkers on overall breast cancer liability. The forest plot in the centre displays the odds ratio of the effect of a SD increase in genetically predicted biomarker concentration on overall breast cancer liability as a square, with error bars representing the 95% CI. In addition to the main analysis based on IVW MR, we include sensitivity analyses based on the weighted median, MR-Egger, MR-PRESSO, and MVMR accounting f [file 12916_2022_2660_MOESM4_ESM.docx]

**Additional file 4: Supplementary Figures.**

**Figure S1. MR forest plot of bone and joint biomarkers on overall breast cancer liability.**

The forest plot in the centre displays the odds ratio of the effect of a SD increase in genetically predicted biomarker concentration on overall breast cancer liability as a square, with error bars representing the 95% CI. In addition to the main analysis based on IVW MR, we include sensitivity analyses based on the weighted median, MR-Egger, MR-PRESSO, and MVMR accounting for known pleiotropic pathways. N. SNPs; number of SNPs. CI; confidence interval. Int. P-value; intercept P-value of MR-Egger. T; Testosterone. BMI; body mass index. An asterisk (*) indicates nominal significance. Two asterisks (**) indicate FDR corrected significance.

**Figure S2. MR forest plot of cancer biomarkers on overall breast cancer liability.**

The forest plot in the centre displays the odds ratio of the effect of a SD increase in genetically predicted biomarker concentration on overall breast cancer liability as a square, with error bars representing the 95% CI. In addition to the main analysis based on IVW MR, we include sensitivity analyses based on the weighted median, MR-Egger, MR-PRESSO, and MVMR accounting for known pleiotropic pathways. N. SNPs; number of SNPs. Int. P-value; intercept P-value. ALP; alkaline phosphatase. T; testosterone. BMI; body mass index. * indicates nominal significance. An asterisk (*) indicates nominal significance. Two asterisks (**) indicate FDR corrected significance.

**Figure S3. MR forest plot of cardiovascular biomarkers on overall breast cancer liability.**

The forest plot in the centre displays the odds ratio of the effect of a SD increase in genetically predicted biomarker concentration on overall breast cancer liability as a square, with error bars representing the 95% CI. In addition to the main analysis based on IVW MR, we include sensitivity analyses based on the weighted median, MR-Egger, MR-PRESSO, and MVMR accounting for known pleiotropic pathways. N. SNPs; number of SNPs. Int. P-value; intercept P-value. BMI; body mass index. An asterisk (*) indicates nominal significance. Two asterisks (**) indicate FDR corrected significance.

**Figure S4. MR forest plot of diabetes biomarkers on overall breast cancer liability.**

The forest plot in the centre displays the odds ratio of the effect of a SD increase in genetically predicted biomarker concentration on overall breast cancer liability as a square, with error bars representing the 95% CI. In addition to the main analysis based on IVW MR, we include sensitivity analyses based on the weighted median, MR-Egger, MR-PRESSO, and MVMR accounting for known pleiotropic pathways. N. SNPs; number of SNPs. Int. P-value; intercept P-value. An asterisk (*) indicates nominal significance. Two asterisks (**) indicate FDR corrected significance.

**Figure S5. MR forest plot of liver biomarkers on overall breast cancer liability.**

The forest plot in the centre displays the odds ratio of the effect of a SD increase in genetically predicted biomarker concentration on overall breast cancer liability as a square, with error bars representing the 95% CI. In addition to the main analysis based on IVW MR, we include sensitivity analyses based on the weighted median, MR-Egger, MR-PRESSO, and MVMR accounting for known pleiotropic pathways. N. SNPs; number of SNPs. Int. P-value; intercept P-value. An asterisk (*) indicates nominal significance. Two asterisks (**) indicate FDR corrected significance.

**Figure S6. MR forest plot of renal biomarkers on overall breast cancer liability.**

The forest plot in the centre displays the odds ratio of the effect of a SD increase in genetically predicted biomarker concentration on overall breast cancer liability as a square, with error bars representing the 95% CI. In addition to the main analysis based on IVW MR, we include sensitivity analyses based on the weighted median, MR-Egger, MR-PRESSO, and MVMR accounting for known pleiotropic pathways. N. SNPs; number of SNPs. Int. P-value; intercept P-value. An asterisk (*) indicates nominal significance. Two asterisks (**) indicate FDR corrected significance.

**Figure S7. MVMR forest plot of lipid biomarkers on overall breast cancer liability.**

The forest plot displays the odds ratio of the effect of a unit increase in genetically predicted biomarker concentration on overall breast cancer liability as a square, with error bars representing the 95% CI. Biomarkers are shown in descending order of significance. An asterisk (*) indicates nominal significance.

**Figure S8. MVMR forest plot of biomarkers, alcohol, and BMI on overall breast cancer liability.**

The forest plot displays the odds ratio of the effect of a unit increase in genetically predicted biomarker concentration on overall breast cancer liability as a square, with error bars representing the 95% CI. Biomarkers are shown in descending order of significance. An asterisk (*) indicates nominal significance.

**Figure S9. MVMR forest plot of sex hormone biomarkers on overall breast cancer liability.**

**
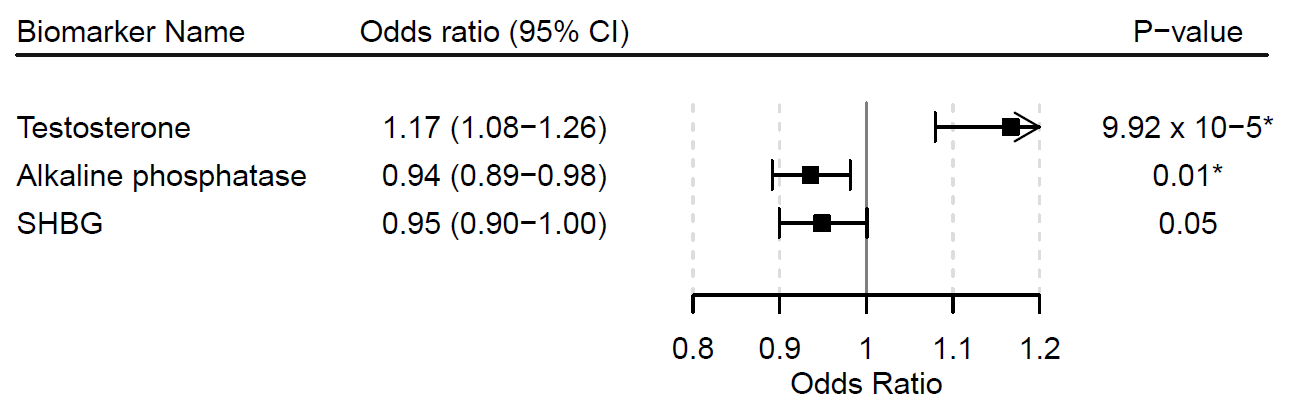
**

The forest plot displays the odds ratio of the effect of a unit increase in genetically predicted biomarker concentration on overall breast cancer liability as a square, with error bars representing the 95% CI. Biomarkers are shown in descending order of significance. An asterisk (*) indicates nominal significance.

**Figure S10. MVMR forest plot of BMI and SHBG on overall breast cancer liability.**

**
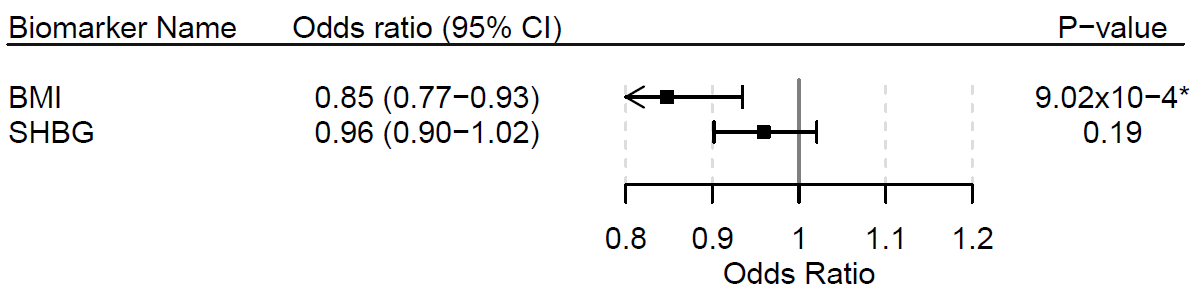
**

The forest plot displays the odds ratio of the effect of a unit increase in genetically predicted biomarker concentration on overall breast cancer liability as a square, with error bars representing the 95% CI. Biomarkers are shown in descending order of significance. An asterisk (*) indicates nominal significance.

**Figure S11. MR forest plot of cardiovascular biomarkers on ER-positive breast cancer liability.**

The forest plot in the centre displays the odds ratio of the effect of a SD increase in genetically predicted biomarker concentration on ER-positive breast cancer liability as a square, with error bars representing the 95% CI. In addition to the main analysis based on IVW MR, we include sensitivity analyses based on the weighted median, MR-Egger, MR-PRESSO, and MVMR accounting for known pleiotropic pathways. N. SNPs; number of SNPs. Int. P-value; intercept P-value. BMI; body mass index. An asterisk (*) indicates nominal significance. Two asterisks (**) indicate FDR corrected significance.

**Figure S12. MR forest plot of bone and joint biomarkers on ER-positive breast cancer liability.**

The forest plot in the centre displays the odds ratio of the effect of a SD increase in genetically predicted biomarker concentration on ER-positive breast cancer liability as a square, with error bars representing the 95% CI. In addition to the main analysis based on IVW MR, we include sensitivity analyses based on the weighted median, MR-Egger, MR-PRESSO, and MVMR accounting for known pleiotropic pathways. N. SNPs; number of SNPs. Int. P-value; intercept P-value. BMI; body mass index. T; testosterone. An asterisk (*) indicates nominal significance. Two asterisks (**) indicate FDR corrected significance.

**Figure S13. MR forest plot of cancer biomarkers on ER-positive breast cancer liability.**

The forest plot in the centre displays the odds ratio of the effect of a SD increase in genetically predicted biomarker concentration on ER-positive breast cancer liability as a square, with error bars representing the 95% CI. In addition to the main analysis based on IVW MR, we include sensitivity analyses based on the weighted median, MR-Egger, MR-PRESSO, and MVMR accounting for known pleiotropic pathways. N. SNPs; number of SNPs. Int. P-value; intercept P-value. ALP; alkaline phosphatase. BMI; body mass index. An asterisk (*) indicates nominal significance. Two asterisks (**) indicate FDR corrected significance.

**Figure S14. MR forest plot of diabetes biomarkers on ER-positive breast cancer liability.**

The forest plot in the centre displays the odds ratio of the effect of a SD increase in genetically predicted biomarker concentration on ER-positive breast cancer liability as a square, with error bars representing the 95% CI. In addition to the main analysis based on IVW MR, we include sensitivity analyses based on the weighted median, MR-Egger, MR-PRESSO, and MVMR accounting for known pleiotropic pathways. N. SNPs; number of SNPs. Int. P-value; intercept P-value. BMI; body mass index. An asterisk (*) indicates nominal significance. Two asterisks (**) indicate FDR corrected significance.

**Figure S15. MR forest plot of liver biomarkers on ER-positive breast cancer liability.**

The forest plot in the centre displays the odds ratio of the effect of a SD increase in genetically predicted biomarker concentration on ER-positive breast cancer liability as a square, with error bars representing the 95% CI. In addition to the main analysis based on IVW MR, we include sensitivity analyses based on the weighted median, MR-Egger, MR-PRESSO, and MVMR accounting for known pleiotropic pathways. N. SNPs; number of SNPs. Int. P-value; intercept P-value. BMI; body mass index. An asterisk (*) indicates nominal significance. Two asterisks (**) indicate FDR corrected significance.

**Figure S16. MR forest plot of renal biomarkers on ER-positive breast cancer liability.**

The forest plot in the centre displays the odds ratio of the effect of a SD increase in genetically predicted biomarker concentration on ER-positive breast cancer liability as a square, with error bars representing the 95% CI. In addition to the main analysis based on IVW MR, we include sensitivity analyses based on the weighted median, MR-Egger, MR-PRESSO, and MVMR accounting for known pleiotropic pathways. N. SNPs; number of SNPs. Int. P-value; intercept P-value. BMI; body mass index. An asterisk (*) indicates nominal significance. Two asterisks (**) indicate FDR corrected significance.

**Figure S17. MR forest plot of bone and joint biomarkers on ER-negative breast cancer liability.**

The forest plot in the centre displays the odds ratio of the effect of a SD increase in genetically predicted biomarker concentration on ER-negative breast cancer liability as a square, with error bars representing the 95% CI. In addition to the main analysis based on IVW MR, we include sensitivity analyses based on the weighted median, MR-Egger, MR-PRESSO, and MVMR accounting for known pleiotropic pathways. N. SNPs; number of SNPs. Int. P-value; intercept P-value. BMI; body mass index. T; testosterone. An asterisk (*) indicates nominal significance. Two asterisks (**) indicate FDR corrected significance.

**Figure S18. MR forest plot of cancer biomarkers on ER-negative breast cancer liability.**

The forest plot in the centre displays the odds ratio of the effect of a SD increase in genetically predicted biomarker concentration on ER-negative breast cancer liability as a square, with error bars representing the 95% CI. In addition to the main analysis based on IVW MR, we include sensitivity analyses based on the weighted median, MR-Egger, MR-PRESSO, and MVMR accounting for known pleiotropic pathways. N. SNPs; number of SNPs. Int. P-value; intercept P-value. BMI; body mass index. T; testosterone. ALP; alkaline phosphatase. An asterisk (*) indicates nominal significance. Two asterisks (**) indicate FDR corrected significance.

**Figure S19. MR forest plot of cardiovascular biomarkers on ER-negative breast cancer liability.**

The forest plot in the centre displays the odds ratio of the effect of a SD increase in genetically predicted biomarker concentration on ER-negative breast cancer liability as a square, with error bars representing the 95% CI. In addition to the main analysis based on IVW MR, we include sensitivity analyses based on the weighted median, MR-Egger, MR-PRESSO, and MVMR accounting for known pleiotropic pathways. N. SNPs; number of SNPs. Int. P-value; intercept P-value. BMI; body mass index. An asterisk (*) indicates nominal significance. Two asterisks (**) indicate FDR corrected significance.

**Figure S20. MR forest plot of diabetes biomarkers on ER-negative breast cancer liability.**

The forest plot in the centre displays the odds ratio of the effect of a SD increase in genetically predicted biomarker concentration on ER-negative breast cancer liability as a square, with error bars representing the 95% CI. In addition to the main analysis based on IVW MR, we include sensitivity analyses based on the weighted median, MR-Egger, MR-PRESSO, and MVMR accounting for known pleiotropic pathways. N. SNPs; number of SNPs. Int. P-value; intercept P-value. An asterisk (*) indicates nominal significance. Two asterisks (**) indicate FDR corrected significance.

**Figure S21.** **MR forest plot of liver biomarkers on ER-negative breast cancer liability.**

The forest plot in the centre displays the odds ratio of the effect of a SD increase in genetically predicted biomarker concentration on ER-negative breast cancer liability as a square, with error bars representing the 95% CI. In addition to the main analysis based on IVW MR, we include sensitivity analyses based on the weighted median, MR-Egger, MR-PRESSO, and MVMR accounting for known pleiotropic pathways. N. SNPs; number of SNPs. Int. P-value; intercept P-value. An asterisk (*) indicates nominal significance. Two asterisks (**) indicate FDR corrected significance.

**Figure S22. MR forest plot of renal biomarkers on ER-negative breast cancer liability.**

The forest plot in the centre displays the odds ratio of the effect of a SD increase in genetically predicted biomarker concentration on ER-negative breast cancer liability as a square, with error bars representing the 95% CI. In addition to the main analysis based on IVW MR, we include sensitivity analyses based on the weighted median, MR-Egger, MR-PRESSO, and MVMR accounting for known pleiotropic pathways. N. SNPs; number of SNPs. Int. P-value; intercept P-value. An asterisk (*) indicates nominal significance. Two asterisks (**) indicate FDR corrected significance.

**Figure S23. MVMR forest plot of lipid biomarkers on ER-positive breast cancer liability.**

The forest plot displays the odds ratio of the effect of a unit increase in genetically predicted biomarker concentration on ER-positive breast cancer liability as a square, with error bars representing the 95% CI. Biomarkers are shown in descending order of significance. An asterisk (*) indicates nominal significance.

**Figure S24. MVMR forest plot of lipid biomarkers on ER-negative breast cancer liability.**

The forest plot displays the odds ratio of the effect of a unit increase in genetically predicted biomarker concentration on ER-negative breast cancer liability as a square, with error bars representing the 95% CI. Biomarkers are shown in descending order of significance. An asterisk (*) indicates nominal significance.

**Figure S25. MVMR forest plot of biomarkers, alcohol, and BMI on ER-positive breast cancer liability.**

The forest plot displays the odds ratio of the effect of a unit increase in genetically predicted biomarker concentration on ER-positive breast cancer liability as a square, with error bars representing the 95% CI. Biomarkers are shown in descending order of significance. An asterisk (*) indicates nominal significance.

**Figure S26.** **MVMR forest plot of biomarkers, alcohol, and BMI on ER-negative breast cancer liability.**

The forest plot displays the odds ratio of the effect of a unit increase in genetically predicted biomarker concentration on ER-negative breast cancer liability as a square, with error bars representing the 95% CI. Biomarkers are shown in descending order of significance. An asterisk (*) indicates nominal significance.

**Figure S27. MVMR forest plot of T, SHBG, and ALP on ER-positive breast cancer liability.**

**
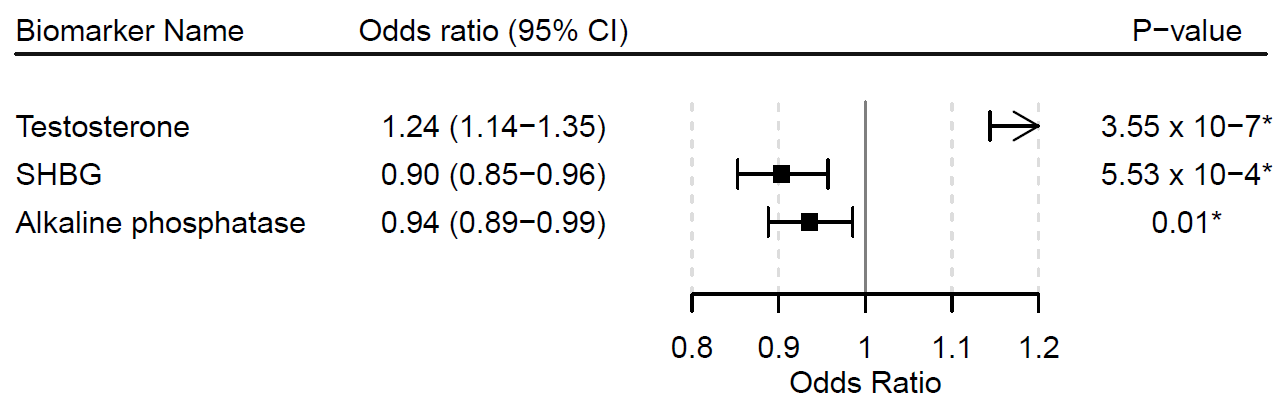
**

The forest plot displays the odds ratio of the effect of a unit increase in genetically predicted biomarker concentration on ER-positive breast cancer liability as a square, with error bars representing the 95% CI. Biomarkers are shown in descending order of significance. An asterisk (*) indicates nominal significance.

**Figure S28. MVMR forest plot of lipid biomarkers on ER-negative breast cancer liability.**


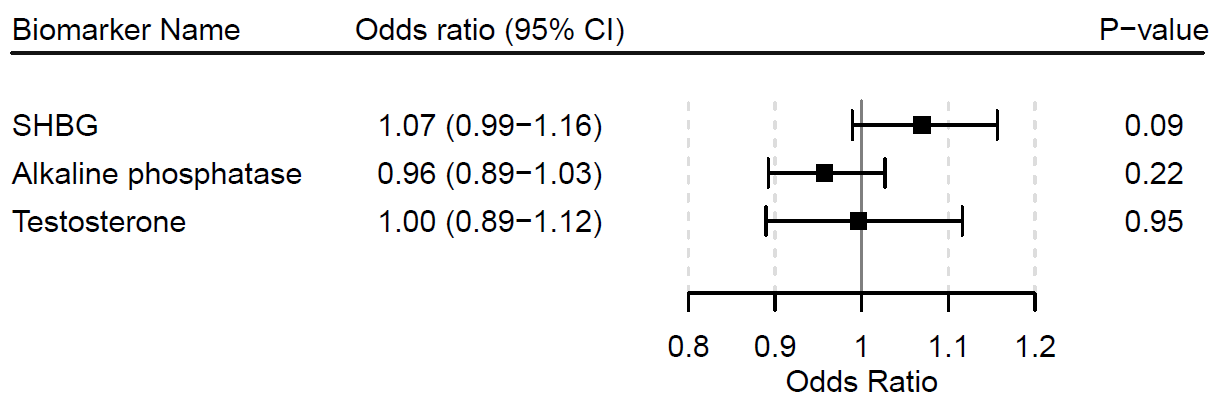


The forest plot displays the odds ratio of the effect of a unit increase in genetically predicted biomarker concentration on ER-negative breast cancer liability as a square, with error bars representing the 95% CI. Biomarkers are shown in descending order of significance. An asterisk (*) indicates nominal significance.

**Figure S29. MVMR forest plot of BMI and SHBG on ER-positive breast cancer liability.**

**
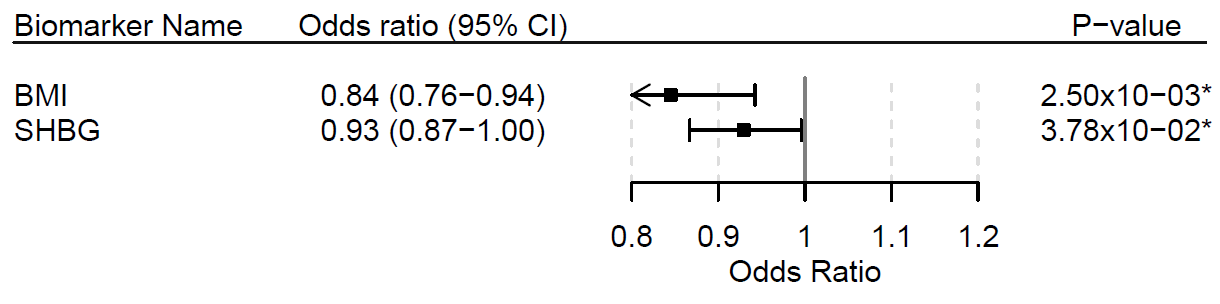
**

The forest plot displays the odds ratio of the effect of a unit increase in genetically predicted biomarker concentration on ER-positive breast cancer liability as a square, with error bars representing the 95% CI. Biomarkers are shown in descending order of significance. An asterisk (*) indicates nominal significance.

**Figure S30. MVMR forest plot of BMI and SHBG on ER-negative breast cancer liability.**


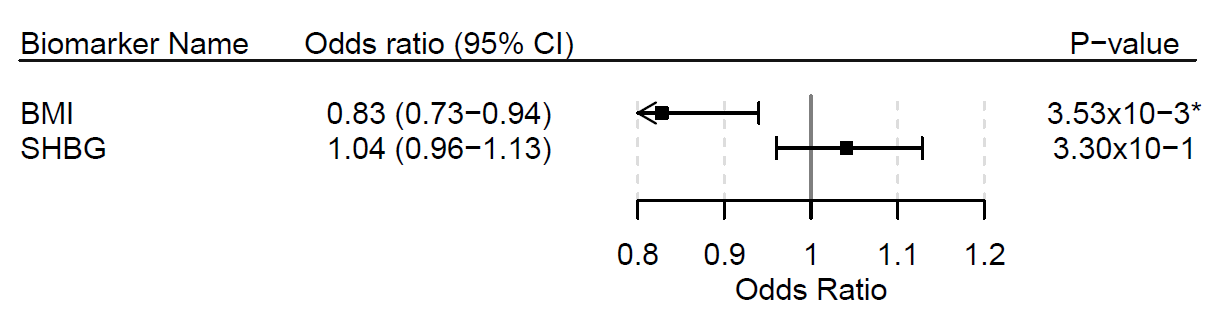


The forest plot displays the odds ratio of the effect of a unit increase in genetically predicted biomarker concentration on ER-negative breast cancer liability as a square, with error bars representing the 95% CI. Biomarkers are shown in descending order of significance. An asterisk (*) indicates nominal significance.
