# Supplementary material for: Identifying and ranking causal biochemical biomarkers for breast cancer: a Mendelian randomisation study
Source: BMC Med. 2022 Nov 23;20:457. doi: 10.1186/s12916-022-02660-2 (PMC9685978; doi:10.1186/s12916-022-02660-2)
Supplement: Supplementary file 5 — Additional file 5: Table S1. Risk and odds of breast cancer per unit increase of each UKB biomarker in the literature and our study. A unit is defined differently in each study. Results in bold font are significant. BC, total breast cancer; PHR, pooled hazards ratio; PRR, pooled risk ratio; POR, pooled odds ratio, SRR, summary risk ratio; preM, pre-menopause; postM, post-menopause; IVW MR, inverse-variance weighted Mendelian randomisation. An asterisk (*) indicates that the ratio method was performed. [file 12916_2022_2660_MOESM5_ESM.docx]

**Additional file 5: Table S1.**

**Table S1. Risk and odds of breast cancer per unit increase in biomarker level in the literature and our study.**

| **Biomarker group** | **Biomarker name** | **Literature findings** | | **Our findings** | | |
| --- | --- | --- | --- | --- | --- | --- |
|  |  | **Risk of overall breast cancer (95% CI)** | **Odds of overall breast cancer from IVW MR (95% CI)** | **Odds of overall breast cancer from IVW MR/ratio method (95% CI)** | **Odds of ER-positive breast cancer from IVW MR/ratio method (95% CI)** | **Odds of ER-negative breast cancer from IVW MR/ratio method (95% CI)** |
| Bone and joint | Alkaline phosphatase | - | - | **0.93 (0.89-0.98)** | **0.94 (0.89-0.99)** | 0.96 (0.90-1.02) |
|  | Calcium | **Meta-analysis PHR: 0.80 (0.66-0.97)** [1] | OR: 0.91 (0.83-1.13) [2] | 1.00 (0.93-1.07) | 1.01 (0.94-1.08) | 1.01 (0.94-1.08) |
|  | Rheumatoid factor | - | - | 0.92 (0.76-1.11)^$^ | 0.89 (0.71-1.12)* | 0.89 (0.71-1.12)* |
|  | Vitamin D | **Prospective cohort HR: 0.95 (0.92-0.98)** [3] | OR: 1.00 (0.98-1.02) [3] | 1.03 (0.96-1.11) | 1.01 (0.91-1.11) | 1.01 (0.91-1.11) |
| Cancer | IGF-1 | **Prospective cohort HR: 1.11 (1.07-1.16)** [4] | **OR: 1.05 (1.01-1.10)** [4] | **1.08 (1.02-1.13)** | **1.07 (1.01-1.14)** | 1.04 (0.97-1.11) |
|  | Oestradiol | **Meta-analysis OR: 2.15 (1.87-2.46)** [5] | - | 0.91 (0.68-1.23)^$^ | 0.96 (0.68-1.37)^$^ | 0.87 (0.51-1.49)^$^ |
|  | SHBG | **Meta-analysis PRR: 0.64 (0.57-0.72)** [6] | OR: 0.96 (0.92-1.00) [7] | 0.98 (0.92-1.05) | 0.95 (0.88-1.03) | 1.07 (0.99-1.16) |
|  | Testosterone | **Meta-analysis OR: 2.04 (1.76-2.37)** [5] | **OR: 1.14 (1.08-1.20)** [8] | **1.12 (1.04-1.21)** | **1.19 (1.09-1.30)** | 0.96 (0.86-1.06) |
| Cardiovascular | Apolipoprotein A | Prospective cohort HR: 1.06 (0.99-1.14) [9] | - | **1.06 (1.02-1.10)** | 1.05 (1.00-1.10) | 1.06 (1.00-1.13) |
|  | Apolipoprotein B | **Prospective cohort HR: 0.92 (0.86-0.99)** [9] | - | 1.00 (0.96-1.05) | 1.00 (0.94-1.05) | 0.97 (0.91-1.04) |
|  | C-reactive protein | Meta-analysis POR: 1.16 (1.06-1.27) [10] | OR: 1.03 (0.94-1.13) [11] | 1.05 (0.99-1.12) | 1.07 (0.99-1.14) | 1.04 (0.96-1.12) |
|  | Cholesterol | Meta-analysis PRR: 0.96 (0.86-1.07) [12] | OR: 1.05 (0.99-1.11) [13] | 1.04 (0.99-1.09) | 1.03 (0.98-1.09) | 1.01 (0.94-1.09) |
|  | HDL cholesterol | Meta-analysis PRR: 0.92 (0.73-1.16) [12] | **OR: 1.12 (1.08-1.16)** [13] | **1.08 (1.04-1.13)** | **1.08 (1.03-1.13)** | **1.08 (1.02-1.15)** |
|  | LDL cholesterol | Meta-analysis PRR: 0.90 (0.77-1.06) [12] | OR: 1.00 (0.96-1.04) [13] | 1.03 (0.98-1.08) | 1.02 (0.96-1.07) | 1.00 (0.93-1.07) |
|  | Lipoprotein A | - | - | 1.01 (0.97-1.05) | 1.01 (0.97-1.05) | 1.03 (0.96-1.11) |
|  | Triglyceride | Meta-analysis PRR: 0.93 (0.86-1.00) [12] | OR: 0.93 (0.85-1.01) [13] | **0.93 (0.88-0.98)** | **0.93 (0.88-0.99)** | **0.92 (0.86-0.99)** |
| Diabetes | Glucose | Meta-analysis SRR: 1.11 (1.00-1.23) [14] | **OR: 1.80 (1.30-2.49)** [15] | 1.01 (0.92-1.11) | 1.01 (0.91-1.12) | 1.06 (0.93-1.19) |
|  | Glycated haemoglobin | - | - | 1.00 (0.95-1.05) | 1.01 (0.95-1.07) | 1.02 (0.95-1.09) |
| Liver | Alanine aminotransferase | - | - | 0.98 (0.89-1.08) | 0.99 (0.88-1.10) | 0.96 (0.85-1.08) |
|  | Albumin | **Case-cohort HR: 0.71 (0.51-0.99)** [16] | - | 0.98 (0.89-1.08) | 0.96 (0.86-1.07) | 1.03 (0.92-1.16) |
|  | Aspartate aminotransferase | - | - | **0.93 (0.88-0.99)** | **0.93 (0.86-1.00)** | 0.94 (0.86-1.02) |
|  | Direct bilirubin | - | - | 1.01 (0.94-1.07) | 1.02 (0.95-1.09) | 0.99 (0.91-1.07) |
|  | Gamma glutamyltransferase | **Meta-analysis PRR: 1.67 (1.12-2.48)** [17] | - | 1.05 (1.00-1.10) | 1.06 (1.00-1.07) | 1.02 (0.96-1.09) |
|  | Total bilirubin | Case-cohort HR: 0.56 (0.31-1.02) [16] | - | 1.01 (0.97-1.06) | 1.02 (0.97-1.07) | 1.01 (0.94-1.07) |
| Renal | Creatinine | - | - | 0.98 (0.93-1.04) | 0.98 (0.93-1.04) | 1.01 (0.95-1.09) |
|  | Creatinine (enzymatic) | - | - | 0.99 (0.74-1.34) | 0.92 (0.70-1.20) | 1.22 (0.55-2.74) |
|  | Cystatin C | - | - | 0.97 (0.91-1.03) | 0.97 (0.91-1.03) | 0.95 (0.87-1.03) |
|  | Microalbumin | - | - | 1.10 (0.79-1.55)^$^ | 1.06 (0.70-1.20)^$^ | 0.86 (0.48-1.60)^$^ |
|  | Phosphate | - | - | 1.06 (0.94-1.20) | 1.06 (0.94-1.04) | 1.05 (0.90-1.22) |
|  | Potassium | - | - | 0.85 (0.44-1.65)^$^ | 0.53 (0.24-1.15)^$^ | 1.28 (0.90-1.06)^$^ |
|  | Sodium | - | - | 1.01 (0.59-1.73) | 1.04 (0.53-2.07) | 0.85 (0.60-1.19) |
|  | Total protein | - | - | 0.95 (0.88-1.03) | 0.96 (0.89-1.03) | 0.91 (0.80-1.03) |
|  | Urate | **Case-control HR: 0.72 (0.53-0.99) (uric acid)** [16] | - | 0.96 (0.91-1.01) | 0.96 (0.91-1.02) | 0.98 (0.90-1.06) |
|  | Urea | - | - | 0.89 (0.79-1.00) | **0.88 (0.78-1.00)** | 0.90 (0.79-1.04) |

A unit is defined differently in each study. Results in bold font are significant. A dollar sign (^$^) indicates that the ratio method was performed. BC, total breast cancer; PHR, pooled hazards ratio; PRR, pooled risk ratio; POR, pooled odds ratio, SRR, summary risk ratio; preM, pre-menopause; postM, post-menopause; IVW MR, inverse-variance weighted Mendelian randomisation.

**References**

1. Wulaningsih W, Sagoo HK, Hamza M, et al (2016) Serum calcium and the risk of breast cancer: Findings from the Swedish AMORIS study and a meta-analysis of prospective studies. Int J Mol Sci 17:1487

2. Papadimitriou N, Dimou N, Gill D, Tzoulaki I, Murphy N, Riboli E, Lewis SJ, Martin RM, Gunter MJ, Tsilidis KK (2020) Genetically predicted circulating concentrations of micro‐nutrients and risk of breast cancer: A Mendelian randomization study. Int J Cancer 668186

3. Ye Y, Yang H, Wang Y, Zhao H (2021) A comprehensive genetic and epidemiological association analysis of vitamin D with common diseases/traits in the UK Biobank. Genet Epidemiol 45:24–35

4. Murphy N, Knuppel A, Papadimitriou N, et al (2020) Insulin-like growth factor-1, insulin-like growth factor-binding protein-3, and breast cancer risk: observational and Mendelian randomization analyses with ∼430 000 women. Annals of Oncology 31:641–649

5. Key T (2015) Steroid hormone measurements from different types of assays in relation to body mass index and breast cancer risk in postmenopausal women: Reanalysis of eighteen prospective studies. Steroids 99:49–55

6. He XY, Liao YD, Yu S, Zhang Y, Wang R (2015) Sex hormone binding globulin and risk of breast cancer in postmenopausal women: A meta-analysis of prospective studies. Hormone and Metabolic Research 47:485–490

7. Dimou NL, Papadimitriou N, Gill D, et al (2019) Sex hormone binding globulin and risk of breast cancer: A Mendelian randomization study. Int J Epidemiol 48:807–816

8. Ruth KS, Day FR, Tyrrell J, et al (2020) Using human genetics to understand the disease impacts of testosterone in men and women. Nat Med 26:252–258

9. Borgquist S, Butt T, Almgren P, Shiffman D, Stocks T, Orho-Melander M, Manjer J, Melander O (2016) Apolipoproteins, lipids and risk of cancer. Int J Cancer 138:2648–2656

10. Guo L, Liu S, Zhang S, Chen Q, Zhang M, Quan P, Lu J, Sun X (2015) C-reactive protein and risk of breast cancer: A systematic review and meta-analysis. Sci Rep 5:1–8

11. Robinson T, Martin RM, Yarmolinsky J (2020) Mendelian randomisation analysis of circulating adipokines and C-reactive protein on breast cancer risk. Int J Cancer 147:1597–1603

12. Ni H, Liu H, Gao R (2015) Serum lipids and breast cancer risk: A meta-Analysis of prospective cohort studies. PLoS One 10:e0142669

13. Beeghly-Fadiel A, Khankari NK, Delahanty RJ, et al (2019) A Mendelian randomization analysis of circulating lipid traits and breast cancer risk. Int J Epidemiol. https://doi.org/10.1093/ije/dyz242

14. Boyle P, Koechlin A, Pizot C, Boniol M, Robertson C, Mullie P, Bolli G, Rosenstock J, Autier P (2013) Blood glucose concentrations and breast cancer risk in women without diabetes: A meta-analysis. Eur J Nutr 52:1533–1540

15. Shu X, Wu L, Khankari NK, et al (2019) Associations of obesity and circulating insulin and glucose with breast cancer risk: A Mendelian randomization analysis. Int J Epidemiol 48:795–806

16. Kühn T, Sookthai D, Graf ME, Schübel R, Freisling H, Johnson T, Katzke V, Kaaks R (2017) Albumin, bilirubin, uric acid and cancer risk: Results from a prospective population-based study. Br J Cancer 117:1572–1579

17. Kunutsor SK, Apekey TA, van Hemelrijck M, Calori G, Perseghin G (2015) Gamma glutamyltransferase, alanine aminotransferase and risk of cancer: Systematic review and meta-analysis. Int J Cancer 136:1162–1170
